# Selective Textile Recycling with Deep Eutectic Solvents: A Mechanistic Framework

**DOI:** 10.3390/polym18161956

**Published:** 2026-08-10

**Authors:** Roderik Plavec, Mária Petková, Slávka Hlaváčiková, Marcela Hricová, Ján Kruželák, Jozef Feranc

**Affiliations:** Institute of Natural and Synthetic Polymers, Faculty of Chemical and Food Technology, Slovak University of Technology, Radlinského 9, 812 37 Bratislava, Slovakia; maria.petkova@stuba.sk (M.P.); slavka.hlavacikova@stuba.sk (S.H.); marcela.hricova@stuba.sk (M.H.); jan.kruzelak@stuba.sk (J.K.); jozef.feranc@stuba.sk (J.F.)

**Keywords:** deep eutectic solvents, textile recycling, selective separation, polymer morphology, solvent–polymer interactions, swelling, depolymerization, polyester/cotton blends

## Abstract

Textile waste is no longer dominated by simple single-polymer materials. Most post-consumer textiles contain combinations of natural and synthetic fibres, elastane, dyes, coatings, finishes, and other additives, which makes selective recycling considerably more difficult. Deep eutectic solvents (DES) offer a promising route for addressing this complexity because their composition and physicochemical properties can be widely tuned. However, the outcome of DES treatment is often discussed mainly in terms of solvent composition or solvent–polymer affinity, although these factors alone cannot explain why similar DES formulations may lead to different responses in different polymeric or textile systems. In this review, DES-assisted textile recycling is examined from a mechanistic polymer-science perspective. The discussion focuses on how polymer morphology, transport accessibility, supramolecular organization, chemical reactivity, and processing conditions jointly determine whether a material undergoes swelling, molecular dissolution, structural destabilization, or chemical degradation. Particular attention is paid to the distinction between these processes, since changes in sample mass, fibre appearance, or crystallinity do not by themselves prove either true polymer dissolution or chain scission. Evidence from cellulose-based fibres, polyesters, polyamides, polyurethanes, elastane-containing materials, and multicomponent textile systems is used to show how different material outcomes may arise from apparently related DES–polymer interactions. The reviewed studies indicate that selectivity in DES-assisted textile recycling should not be treated as a fixed property of the solvent or of the polymer alone. It is more appropriately understood as the result of a coupled and time-dependent interaction between the DES medium, polymer morphology, textile architecture, and processing conditions. The mechanistic framework proposed here provides a basis for comparing reported DES-based recycling strategies, identifying the experimental evidence needed to support mechanistic claims, and guiding the rational selection of DES composition, process conditions, and recovery pathways for complex textile waste.

## 1. Introduction

Increasing consumption of textile products, the expansion of fast-fashion business models, and the shorter service life of garments have led to a sharp increase in textile waste generation over recent decades, while material recycling remains highly limited [1,2,3,4,5]. Despite the growing emphasis on circular-economy principles, most end-of-life textiles are still landfilled or incinerated with energy recovery, resulting in the loss of valuable polymer feedstocks and further environmental burdens [2,4,5].

One of the main reasons for the low recycling rate is the inherent complexity of modern textile materials. In addition to combinations of natural and synthetic fibres, textiles may contain elastane components, polyurethanes, dyes, surface finishes, and other functional additives, all of which substantially complicate their selective processing [5,6,7]. Effective recycling therefore requires not only polymer degradation or dissolution, but above all the targeted separation of individual components while preserving their material value to the greatest possible extent.

Current textile-recycling strategies include mechanical, chemical, and solvent-based approaches. Mechanical recycling is technologically simple but is largely limited to relatively clean, single-material streams and often leads to the progressive deterioration of recycled-fibre properties [4,8,9]. Chemical recycling enables the recovery of polymer feedstocks through depolymerization, but its application to complex textile blends remains constrained by the need for selective removal or transformation of individual polymer components [10,11,12,13]. Consequently, increasing attention has been directed toward solvent-based technologies that rely on selective interactions with individual polymers and may provide a promising alternative to conventional degradative processes [8,14,15,16,17,18,19,20,21].

Among the most extensively investigated solvent systems are deep eutectic solvents (DES). Their chemical composition can be readily varied, enabling substantial tuning of physicochemical properties such as polarity, acid–base character, viscosity, and hydrogen-bonding capacity. This compositional flexibility provides a broad range of systems potentially suitable for interaction with different polymers [20,21,22,23,24,25,26].

Most experimental studies describe the selective behaviour of individual DES systems primarily in terms of solvent composition or solvent–polymer affinity. However, this approach often fails to explain why chemically similar DES formulations may lead to markedly different outcomes in apparently similar polymeric or textile systems. Available evidence further suggests that the resulting selectivity also depends on polymer morphology, solvent transport into the polymer matrix, supramolecular organization, and the time-dependent evolution of these factors during interaction [21,23,27,28,29,30,31,32,33,34].

Most existing review articles organize published studies primarily according to DES composition, polymer type, or specific application areas. Considerably less attention has been devoted to the common mechanisms governing the selective behaviour of DES across different polymer systems and to the interplay among transport phenomena, polymer morphology, supramolecular interactions, and chemical processes. The absence of such an integrated perspective complicates comparison of published results and identification of the factors determining successful selective separation.

The aim of this review is therefore not to provide another overview of deep eutectic solvents used in textile recycling, but to critically analyze the mechanisms responsible for their selective behaviour toward polymeric materials. Based on the available literature, this work integrates concepts from polymer physics, transport phenomena, supramolecular interactions, and chemical reactivity into a unified mechanistic interpretive framework that enables more consistent interpretation of experimental results reported for different DES–polymer systems. It also provides a common basis for the polymer-specific sections that follow.

This review was conceived as a critical, mechanistically oriented narrative review rather than as a formal systematic review. Relevant literature was identified primarily through the Web of Science Core Collection and Scopus databases and supplemented by backward and forward citation tracking of key publications. The literature search was updated through 31 July 2026. Searches combined terms related to deep eutectic solvents with textile-relevant polymers, recycling, swelling, dissolution, degradation, rheology, and solvent regeneration. Priority was given to studies providing experimental information on the polymer, solvent composition, processing conditions, and material response, whereas unrelated DES applications and studies lacking sufficient experimental detail were excluded.

**Figure 1:**
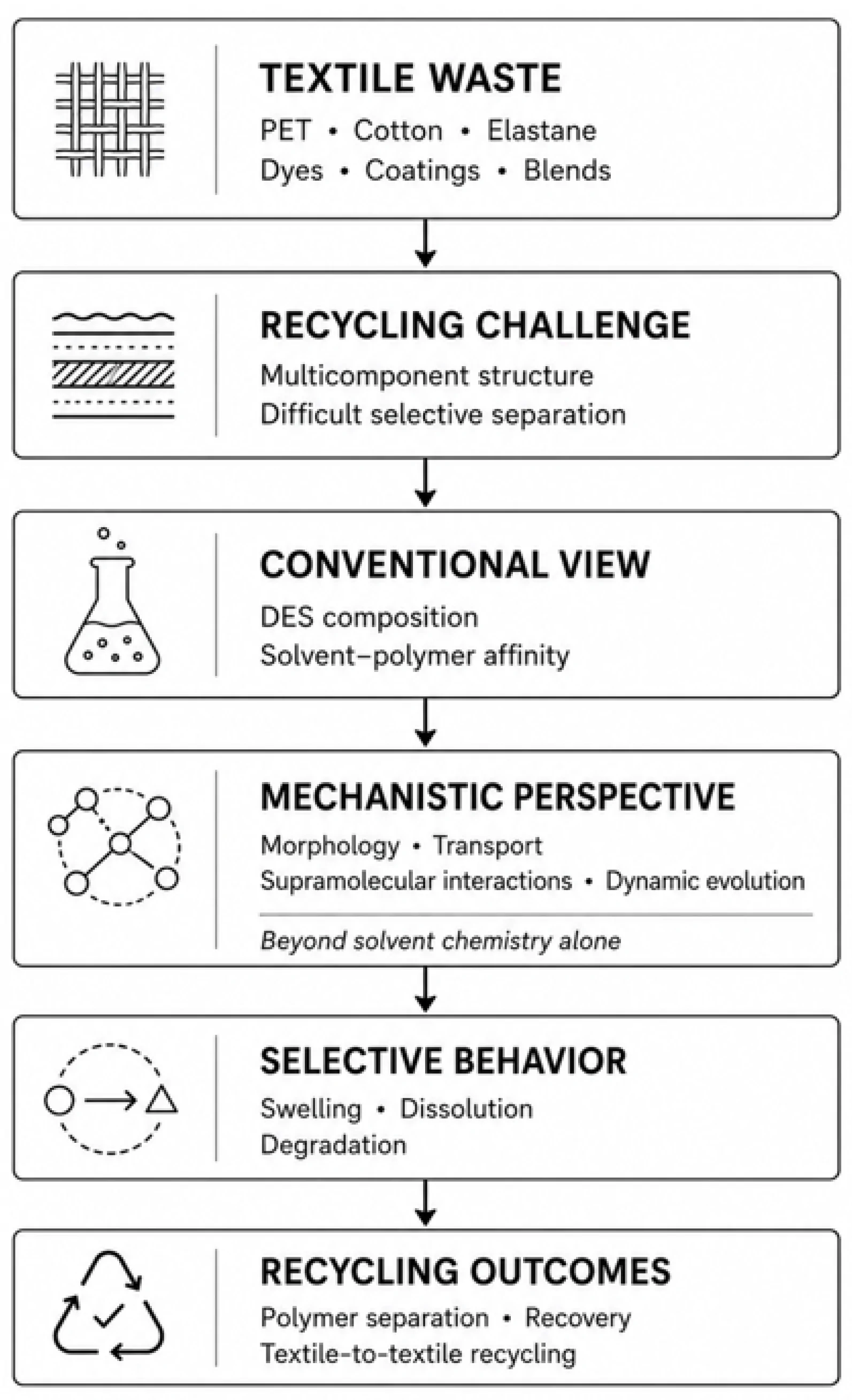
Conceptual framework of selective textile recycling using deep eutectic solvents.

Figure 1 schematically summarizes the main approaches used in textile recycling, their interrelationships, and the position of the mechanistic perspective discussed in this review.

As illustrated in Figure 1, the individual recycling strategies rely on different technological principles but share the challenge of selectively separating polymer components from complex textile materials. Table 1 summarizes the main conceptual differences between the conventional interpretation of DES–polymer interactions and the mechanistic perspective proposed in this work.

## 2. Textile Recycling Technologies and Deep Eutectic Solvents

### 2.1. Current Approaches to Textile Recycling

Current textile recycling strategies can be broadly categorized into mechanical, chemical, and solvent-based approaches, whereas thermochemical and biological processes represent complementary routes for specific material streams. Their practical value, however, depends not only on the conversion achieved or product yield, but primarily on their ability to process real, heterogeneous textile waste while preserving the highest possible material value of the individual polymer components. The selection of an appropriate strategy therefore depends on the chemical composition of the textile, the degree of contamination, the presence of surface finishes, fibre architecture, and the desired final product [4,5,6,7,29,30].

Mechanical recycling is the most widely applied route for processing textile waste. It generally involves sorting, cutting, shredding, fibre opening, carding, and reprocessing of the recovered fibres without substantial alteration of the chemical structure of the polymers. It is particularly suitable for relatively clean and homogeneous material streams, especially post-industrial waste. However, in post-consumer textiles, the presence of blended fibres, dyes, surface finishes, and contaminants substantially limits the quality of the resulting material. Repeated mechanical processing further leads to fibre shortening and often restricts the recovered material to applications such as insulation, fillings, and technical nonwoven products, which represent an important outlet for mechanically recycled textile fibres [5,6,7,29,35].

Chemical recycling enables the targeted conversion of polymer structures into monomers, oligomers, or other chemically valuable intermediates. For PET, the most extensively developed routes include glycolysis, hydrolysis, and methanolysis, which allow the recovery of compounds suitable for subsequent repolymerization [11,12,13,36]. A key advantage of chemical recycling is its potential to recover material value from fibres that are no longer suitable for direct mechanical processing. However, in blended textiles, the selectivity of the reaction toward individual polymer components must be carefully controlled, as accompanying fibres, dyes, or surface finishes may complicate depolymerization, contaminate the products, or undergo irreversible damage during processing [35,37,38,39].

A distinct group of recycling strategies is based on the selective dissolution of one polymer component while preserving the remaining constituents of the textile system. Unlike depolymerization routes, their primary objective is not chain scission, but the controlled separation of polymer phases, enabling subsequent recovery of either the dissolved or undissolved material. The potential of such approaches has been demonstrated, for example, in the separation of cotton/polyester blends using ionic liquids, where one polymer fraction can be dissolved while the other is retained in the solid state [40,41]. The practical feasibility of solvent-based recycling, however, is strongly influenced by the accessibility of polymer domains, the rate of medium penetration, the need for solvent regeneration, the purity of the recovered fractions, and the capacity of the process to handle real blended textiles [35,40,41].

Thermochemical processes, such as pyrolysis or gasification, enable the treatment of poorly sorted and highly contaminated waste streams, but generally result in the loss of the original polymer structure and fibre-specific material value. In contrast, biological and enzymatic approaches offer the potential for more selective transformations; however, their broader implementation remains limited by slower kinetics, restricted accessibility of polymer bonds, and the need for suitable reaction conditions [29,30,35].

From the perspective of selectively recycling complex textile systems, no universal technological route is available. Mechanical recycling is particularly suitable for clean material streams, chemical depolymerization enables monomer recovery but requires careful control of reactivity toward coexisting components, whereas solvent-based approaches offer the possibility of directly separating polymer fractions. The ability to control the interaction between the processing medium and individual polymer domains is therefore a key prerequisite for more efficient treatment of multicomponent textile waste. The main limitations of the individual approaches with respect to selective separation are summarized in Table 2.

### 2.2. Deep Eutectic Solvents: Principles, Relevant Properties, and Practical Considerations for Textile Recycling

Solvent-based recycling strategies require media capable of selectively interacting with individual polymer components without necessarily causing the complete degradation of the textile system. In this context, deep eutectic solvents (DES) have received considerable attention in recent years, as they can function as solvents, reaction media, catalytically active components, or combinations thereof in the processing of polymeric materials [10,20,26,42,43,44]. Their relevance to textile recycling arises primarily from the possibility of varying their chemical composition and thereby tailoring their properties to the characteristics of the material being processed.

DES can generally be described as eutectic liquid mixtures in which specific intermolecular interactions between the components lead to a substantial depression of the melting temperature relative to those of the pure constituents. In many commonly used systems, one component acts as a hydrogen-bond acceptor (HBA), such as choline chloride, whereas the other acts as a hydrogen-bond donor (HBD), such as urea, ethylene glycol, glycerol, or an organic acid. However, the resulting liquid structure is determined not only by the presence of an HBA and HBD, but also by their chemical nature, molar ratio, potential complexation, water content, and temperature [20,21,22,23]. Related literature also refers to low-transition-temperature mixtures (LTTMs) and natural deep eutectic solvents (NADES), highlighting the broader chemical variability of low-melting mixtures and the potential use of naturally derived components in their formulation [25,45,46].

This compositional variability enables the adjustment of DES viscosity, polarity, hydrophobicity, acid–base character, hydrogen-bonding capacity, and thermal stability. From the perspective of textile recycling, these properties do not act independently. For example, lower viscosity may improve handling and facilitate transport of the medium into the textile structure, whereas a suitable acid–base or Lewis-acidic character may promote the activation of specific chemical bonds. Likewise, water content may reduce viscosity while simultaneously reorganizing the supramolecular network of the DES, altering the solvation properties of the medium and affecting its reactivity toward the polymer matrix [20,21,23,26].

Compared with conventional organic solvents, DES represent a potentially advantageous platform because of their relatively simple preparation, the broad availability of possible components, and the possibility of formulating systems with different affinities toward hydrophilic and hydrophobic polymer components. This flexibility is particularly relevant for textile waste, in which cellulosic fibres, polyesters, elastane, or polyurethane components, dyes, and surface finishes may coexist within a single material. DES should therefore not be regarded merely as substitutes for conventional solvents, but rather as potentially tunable interaction media whose function may range from physical swelling and selective dissolution to catalytically assisted chemical transformation of the polymer [10,20,26,44].

These potential advantages do not imply that every DES is automatically suitable for practical or environmentally favourable textile recycling. The actual applicability of a system depends on its viscosity, the required processing temperature, the DES-to-textile ratio, stability during treatment, the potential for medium regeneration, the purity of the recovered polymer fractions, and the energy demand associated with subsequent purification or product separation. Similarly, the designation of a system as “green” cannot be inferred solely from its classification as a DES; it must be evaluated in relation to its specific composition, the toxicity of the components used, the recyclability of the medium, and the overall process configuration [20,21,22,23].

DES should also be considered in relation to established cellulose solvent systems such as N-methylmorpholine N-oxide (NMMO) and ionic liquids. For denim-derived cellulose in NMMO, concentrations of 4–8 wt% were examined, and the 6 wt% solution at 80 °C showed the most favourable balance of viscous and elastic behaviour within the investigated range [47]. The ionic liquid [DBNH][OAc] enabled selective dissolution of cotton from cotton/PET textiles and produced cellulose solutions containing 6.5 or 10.5 wt% cellulose that were directly processed by dry-jet wet spinning after PET removal [48]. A recent ChOH/urea/zinc glycinate DES dissolved 12 wt% microcrystalline cellulose at 65 °C and maintained more than 80% cellulose-solubilization efficiency after three cycles [49]. These examples demonstrate that solvent performance should be assessed not only by whether dissolution occurs, but also by the attainable polymer concentration, rheological behaviour, regeneration route, and intended downstream process.

From a mechanistic perspective, it is therefore essential to recognize that the chemical formulation of a DES alone does not determine the outcome of its interaction with a polymer. The final response arises from the combined effects of DES properties, polymer-matrix morphology, transport accessibility of individual domains, and process conditions. The tunability of DES is thus their principal advantage, but it is also the reason why the results of individual studies cannot be directly compared solely on the basis of the name or chemical composition of the solvent used. The key physicochemical properties of DES relevant to textile recycling are summarized in Table 3.

### 2.3. Current Limitations and the Need for a Mechanistic Framework

Despite the growing number of reported DES systems, their comparison in textile recycling remains challenging. The outcomes of individual studies are often assessed using final parameters such as polymer conversion, yield of degradation products, mass loss, or the fraction of recovered polymer. Although such data are essential for evaluating process performance, they may not, by themselves, unambiguously reveal the mechanism operating in the system. For example, DES-assisted processing of PET/cotton textiles may involve selective polyester glycolysis, alkaline hydrolysis, molecular dissolution of one polymer phase, or a combination of several partial processes [8,14,15,16].

A further important issue is the inconsistent use of the terms *swelling*, *dissolution*, and *degradation*. An increase in volume, fibre softening, mass loss, or the apparent disappearance of one textile component may not be sufficient to distinguish unambiguously between medium penetration into the polymer matrix, molecular dissolution of the polymer, and chemical chain scission. A more reliable interpretation therefore requires the combined assessment of molar mass, chemical structure, crystallinity, morphology, and, where relevant, the composition of the resulting products. Without such complementary characterization, mechanistic conclusions based solely on conversion or mass balance should be regarded as limited [8,14,15,16].

Comparison of published results is further complicated by the substantial variability in experimental conditions. DES systems differ in composition, component molar ratio, water content, viscosity, acid–base character, and reaction temperature, whereas polymeric materials may differ in crystallinity, chain orientation, fibre dimensions, processing history, or degree of prior degradation. The broader literature on DES in polymer systems has also developed across diverse application areas, ranging from membrane materials to the preparation and modification of polymeric systems [27,28]. In real post-consumer textiles, these factors are further compounded by dyes, surface finishes, laminates, elastane components, compositional heterogeneity, and varying degrees of contamination, all of which substantially limit the direct transferability of results obtained using model polymers or simple blends [5,6,29,30].

These considerations indicate that empirical screening of new DES formulations remains important; however, the resulting observations should be interpreted within the broader context of solvent–polymer interactions and polymer structure. The aim of the following section is therefore not to establish a universal predictive model, but to provide a mechanistic interpretive framework linking transport accessibility of the polymer matrix, supramolecular interactions, structural reorganization, and the subsequent processes of *swelling*, *dissolution*, and *degradation*. Such a perspective enables more systematic comparison of different DES–polymer systems and helps explain why similar formulations or apparently similar textile materials may not lead to the same selective outcome.

## 3. Mechanistic Perspective for Selective Separation

For selective textile recycling, final conversion, mass loss, or the yield of an isolated fraction are often the most accessible experimental outputs, but they rarely explain what actually happened to the polymer. Similar mass losses may originate from swelling, fragmentation, dissolution, or chemical degradation, whereas comparable DES formulations may produce different responses in polymers with different morphology, accessibility, or processing history. A meaningful interpretation of DES–polymer systems therefore has to separate solvent–polymer chemical affinity from transport accessibility, supramolecular reorganization, and possible chemical transformation of the polymer chains [8,14,15,16,20,23,27,28].

In the framework adopted here, selectivity is treated as the outcome of a sequence of coupled events rather than as an immediate consequence of solvent choice. Contact between the DES and the material surface is followed by penetration into transport-accessible regions of the polymer. This early stage may involve swelling, changes in local free volume, disruption or rearrangement of hydrogen bonds and other intermolecular interactions, and the gradual opening of additional polymer domains to the medium. Only after these initial events does the system move, depending on polymer chemistry, DES composition, and processing conditions, toward molecular dissolution, partial structural destabilization, or polymer-chain scission [21,23,31,32,33,34,50,51,52,53].

The purpose of this mechanistic perspective is not to provide a universal predictive model for all DES–polymer systems. Individual systems still require direct experimental verification. The value of the framework is instead in offering a common way to read and compare published results. In the following subsections, DES-assisted selectivity is therefore discussed through four connected levels: (i) polymer morphology and transport accessibility; (ii) the distinction between swelling, dissolution, and degradation; (iii) the time-dependent evolution of the interaction; and (iv) the polymer-, solvent-, process-related, and textile-structural factors that ultimately control the observed outcome.

### 3.1. Polymer Morphology and Transport Accessibility

The transport accessibility of the polymer matrix represents the first physical factor determining whether a DES can effectively reach the functional groups or reactive bonds of a polymer. In semicrystalline polymers, the polymer structure is not homogeneous. Crystalline regions are generally more densely packed and less accessible to penetration by a liquid medium, whereas amorphous regions provide greater free volume and higher segmental mobility. These less ordered domains therefore often constitute the initial sites of sorption and solvent ingress into the polymer matrix [31,32,33,34,50,51,52,53].

The extent of such accessibility depends not only on the overall fraction of crystalline and amorphous regions, but also on chain orientation, local packing density, free volume, and the mobility of polymer segments at a given temperature. In polymers processed below or near the glass-transition temperature, chain reorganization during medium sorption may be restricted, whereas greater segmental mobility may facilitate further solvent penetration and subsequent structural changes. Transport accessibility should therefore not be regarded as an inherent constant property of a polymer, but rather as the result of its morphology and the prevailing conditions of interaction with the processing medium [31,32,33,34,50,51,52].

At the simplest level, solvent transport into a polymer may be described by Fick’s first law:

(1) $$J=-D\frac{\partial c}{\partial x}$$

where J is the solvent flux, D is the effective diffusion coefficient, and ∂c/∂x is the concentration gradient. In heterogeneous textile fibres, however, D should be regarded as a morphology-dependent effective parameter rather than as a constant, because crystallinity, pore structure, chain orientation, swelling, and polymer relaxation may change during penetration. Direct diffusion coefficients are rarely reported for textile-relevant solvent systems; therefore, solvent uptake and swelling kinetics are often used as indirect indicators of transport accessibility. Qian et al. reported that cellulose pulp showing the highest NMMO-retention (729.5%) after 20 min of swelling in 70 wt.% NMMO at 80 °C also exhibited the shortest subsequent dissolution time (20 min), demonstrating the close relationship between fibre structure, solvent uptake, and dissolution behaviour [54].

The importance of initial structural accessibility is well illustrated by the treatment of cotton using a DES system. Liu et al. [55] reported that the disintegration of cotton fibres is limited by the strong hydrogen-bonding network of cellulose, as well as by surface layers and the heterogeneous structure of the fibre. Following microwave-assisted DES pretreatment and subsequent ultrasonication, cellulose nanocrystals were obtained in high yield. This result does not imply that the DES alone completely dissolved cellulose; rather, it demonstrates that the initial disruption of intermolecular interactions and increased accessibility of the fibre structure can substantially alter the subsequent response of the material [55].

The situation is even more complex for textile fibres because their morphology is strongly influenced by processing history. Fibre spinning, drawing, and thermal treatment may result in high chain orientation, increased crystallinity, or radial differences between the surface and inner regions of the fibre. Transport of the medium may therefore not occur uniformly across the entire fibre cross-section, but may instead be localized in surface, less ordered, or defect-rich regions. Additional barriers may be created by dyes, surface finishes, laminates, or multilayer textile architectures, which can restrict direct contact between the DES and the polymer matrix [35,40,56,57,58,59,60,61].

This distinction is particularly important for PET. Enking et al. [35] emphasized that, from a recycling perspective, PET fibres cannot be considered equivalent to PET bottles because they differ in crystallinity, chain orientation, purity, and the presence of accompanying textile components. Results obtained for neat PET films or packaging materials therefore cannot be directly transferred to real PET textiles. High fibre orientation and crystallinity may limit medium penetration even when the DES or reaction medium has favourable chemical affinity toward ester groups [35].

Studies on DES-catalyzed PET glycolysis likewise highlight the importance of the interplay between chemical and transport-related factors. Sert et al. [44] showed that changes in the DES system, its viscosity, and its basic character led to differences in PET conversion and BHET yield. Although these results do not directly separate the contributions of diffusion, sorption, and chemical reactivity, they confirm that the final yield cannot be interpreted solely as a consequence of catalytic activity. In real polymeric materials, the effect of the chemical composition of the medium is necessarily coupled with its ability to penetrate the polymer phase and maintain sufficient contact with accessible reactive sites [44,58].

Similarly, Choi and Choi [62] demonstrated rapid removal of PET from PET/cotton textiles using a glycerol/choline chloride system containing NaOH under microwave heating. The process was evaluated using textiles with different PET-to-cotton ratios as well as a commercial knitted fabric, while the condition of the remaining textile structure was characterized by FTIR, XRD, SEM, and TGA. Although this study did not directly determine diffusion coefficients, it shows that the resulting response of a textile system arises from the combined effects of medium composition, thermal input, chemical reactivity, and the specific fibre architecture, rather than from solvent affinity toward PET alone [62].

In polymers in the glassy state, medium transport may also be coupled with time-dependent relaxation and deformation of the polymer matrix induced by swelling stresses. In classical polymer–penetrant systems, such behaviour has been described as Case II diffusion, in which the progression of the penetrating medium is governed not only by its diffusivity but also by the rate of relaxation of the polymer structure [63]. In multilayer or laminated materials, this coupling between penetration and structural response is further complicated by interfaces between individual layers and differences in the accessibility of individual polymer phases, which may further affect the selective action of DES [64].

Such heterogeneity means that the first experimentally observed material response may not represent the behaviour of the entire polymer phase. Surface softening, localized swelling, or changes in fibre appearance may reflect interactions limited to more accessible domains, whereas more crystalline or highly oriented regions may remain largely unaffected under the same exposure conditions. When evaluating DES–polymer systems, it is therefore important to distinguish between surface-limited interactions and processes affecting the entire volume of the polymer matrix [31,32,33,34,50,51,52,53].

Overall, polymer morphology determines not only the rate of DES penetration, but also which polymer domains become accessible during the initial stage of interaction. This initial transport heterogeneity may subsequently influence the course of swelling, supramolecular reorganization, dissolution, or chemical degradation. The main morphological factors that may modify the accessibility of the polymer matrix to DES are summarized in Table 4.

### 3.2. Swelling, Dissolution, and Degradation

**Figure 2:**
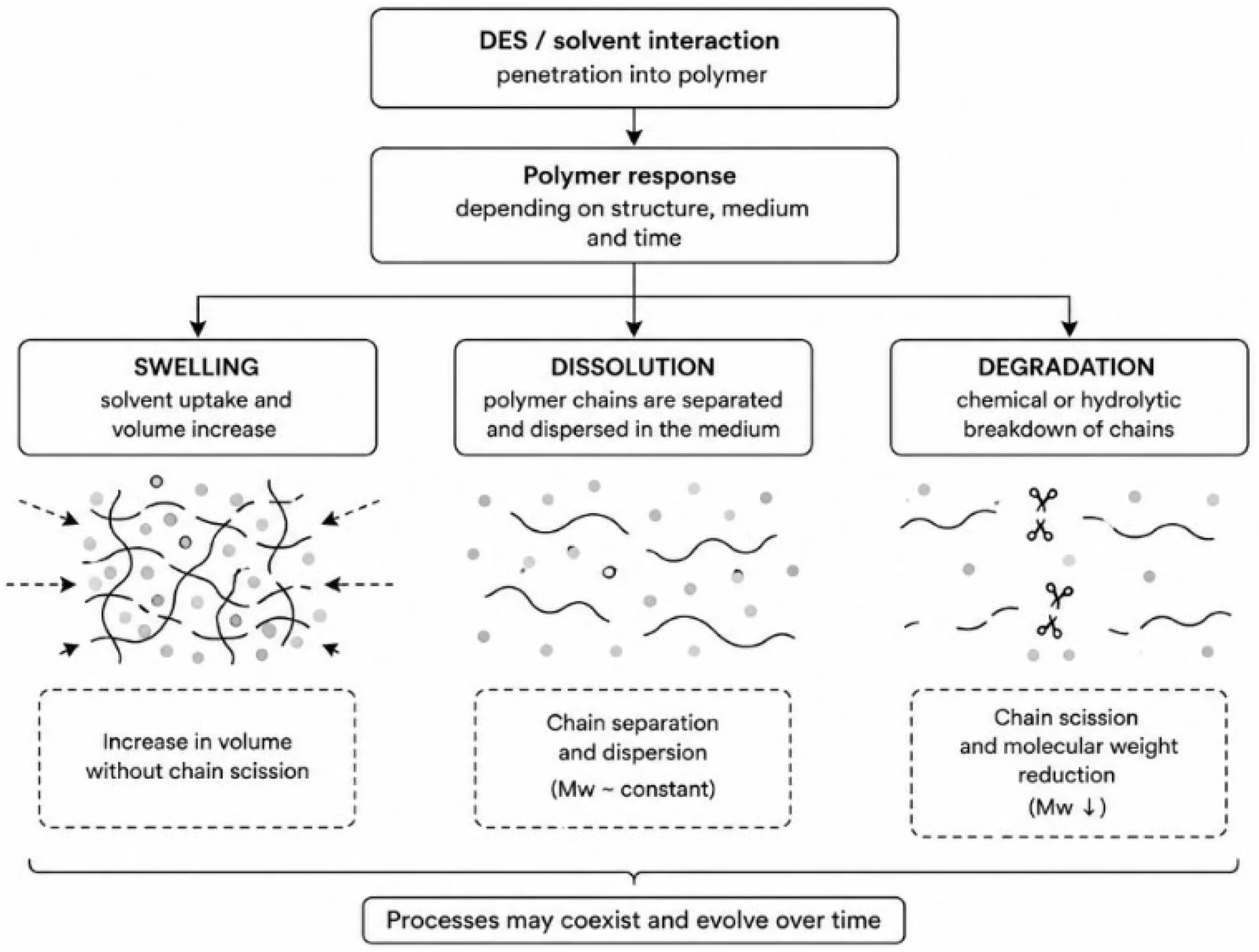
Simplified distinction between swelling, dissolution, and degradation pathways during DES/polymer interaction.

Following the penetration of a DES or another processing medium into the polymer matrix, the material may undergo a series of successive physical and chemical changes. The processes most commonly distinguished in the literature are *swelling*, *dissolution*, and *degradation*. However, in real DES–polymer systems, these processes cannot always be regarded as strictly separate mechanisms. Rather, they represent different levels of polymer response, the relative importance of which depends on the accessibility of the polymer matrix, the intensity of interaction with the medium, the chemical reactivity of the system, and the exposure time [17,18,21,23,27]. The conceptual distinction between these three possible polymer responses is schematically summarized in Figure 2.

*Swelling* refers to the penetration of a medium into the polymer matrix, accompanied by an increase in volume, softening, or local structural reorganization, without demonstrated cleavage of polymer chains. In fibrous materials, this process may be limited to surface or less ordered regions, while the remaining part of the fibre remains relatively unaffected. Such partial destabilization may be technologically relevant even in the absence of complete dissolution, as disruption of hydrogen bonds or an increase in local free volume can render the polymer more accessible to subsequent mechanical, chemical, or solvent-based treatment. In cotton fibres, Liu et al. [55] showed that DES pretreatment promoted disruption of the strong hydrogen-bonding network and subsequent ultrasonic fibrillation, with the primary role of the medium being to increase the accessibility of the cellulosic structure rather than to induce immediate and complete polymer dissolution.

In contrast, *dissolution* should be used to describe a state in which polymer chains are dispersed within the solvent medium and the original supramolecular organization of the polymer phase is no longer retained. In practice, however, the apparent disappearance of fibres, mass loss, or the formation of a clear solution do not by themselves provide sufficient evidence of molecular dissolution. Such observations may also result from fragmentation, localized degradation, or the formation of a fine suspension. A more convincing assessment of dissolution therefore requires a combination of visual observations with filtration, polymer regeneration, rheological characterization, and, where possible, determination of the molar mass of the regenerated fraction. For processes intended to regenerate fibres by spinning, confirmation of dissolution is only a first requirement. The attainable polymer concentration and the balance between viscous and elastic behaviour must also permit stable extrusion and filament formation. No universal target viscosity can be defined independently of polymer molar mass, temperature, shear rate, and the specific spinning configuration [47,48]. An example of selective PET dissolution from a PET/cotton textile blend was reported by Depope et al. [15], who observed complete dissolution of PET while preserving the cotton component and achieving a high degree of polyester recovery.

*Degradation* involves the chemical cleavage of covalent bonds within polymer chains, manifested by a decrease in molar mass, the formation of oligomers or monomers, and/or changes in the chemical structure of the material. In polyester systems, this may involve hydrolytic or glycolytic cleavage of ester bonds, whereas in polyurethanes it may involve cleavage of urethane linkages. Zhang et al. [8] showed that a combination of ChCl:ethylene glycol and NaOH enabled selective degradation of PET in a PET/cotton system while preserving the cellulosic fibres. In polyurethane, Kaur et al. [65] demonstrated that a lactic acid:ZnCl_2_ system resulted not only in dissolution of the material, but also in the formation of low-molar-mass products, cleavage of urethane bonds, and the generation of new functional groups. These findings demonstrate that a process initially described as dissolution may simultaneously involve substantial chemical transformation of the polymer.

The boundaries between these processes are therefore often dynamic. Initial swelling may increase the transport accessibility of the polymer, weaken intermolecular interactions, and create conditions for subsequent dissolution or degradation. Conversely, chemical degradation may reduce molar mass and alter crystallinity, thereby further accelerating medium transport into initially less accessible regions. During the selective dissolution of cotton from a PET/cotton/PU system, Zeng et al. [66] observed a gradual progression from intact and locally wetted fibres through swelling and fragmentation to a homogeneous transparent medium with no remaining intact cellulosic fibres. This type of time-resolved observation is important because it demonstrates that the final process outcome may not reflect the initial stage of interaction.

It follows that the terminological distinction between *swelling*, *dissolution*, and *degradation* is not merely a formal issue. Misinterpretation may lead to overestimation of the dissolution capacity of a DES, incorrect assessment of polymer-value preservation, or a lack of comparability among studies. Reliable mechanistic assessment therefore requires the combined use of methods monitoring changes in mass, morphology, crystallinity, chemical structure, molar mass, and the composition of reaction products. The main experimental indicators and their cautious interpretation are summarized in Table 5.

The processes discussed in this subsection are not temporally static. The following section therefore examines how transport, structural reorganization, and chemical transformation of the polymer may interact during a single exposure period and give rise to the dynamic evolution of selective separation.

### 3.3. Dynamic Evolution of Selective Separation

The interaction between a DES and a polymer rarely remains fixed during exposure. The starting morphology of the polymer defines which regions are first accessible to the medium, but the medium itself may then change this accessibility. Local swelling, disruption of intermolecular interactions, or partial decrystallization can open regions that were initially less available for further interaction. Selective separation should therefore be viewed as a process that may pass through several structural and chemical stages, with their relative importance controlled by polymer structure, DES composition, and processing conditions [21,23,27,31,32,33,34,50,51,52,53].

This evolution does not follow a single universal pathway. In some systems, the interaction stops at surface swelling or limited supramolecular reorganization. In others, the same initial penetration step may be followed by molecular dissolution or chemical degradation. The final state of the material can therefore be misleading if it is interpreted without considering the preceding stages. A polymer that is fully dissolved or degraded after prolonged exposure may have first undergone only local medium uptake and gradual structural destabilization. Conversely, a change in fibre appearance or partial mass loss does not prove that the whole polymer phase was equally accessible during treatment.

Cellulosic systems provide a clear example of this time-dependent response. Liu et al. [55] showed that DES pretreatment of cotton fibres weakened the hydrogen-bonding network and increased the accessibility of cellulose for subsequent ultrasonic fibrillation. The important point in this case was not immediate cellulose dissolution, but the gradual preparation of the fibre structure for further transformation. A similar sequence was observed by Zeng et al. [66] during the selective treatment of PET/cotton/PU textiles. The cotton component evolved from intact fibres with slight surface wetting through swelling and fragmentation to complete removal of intact cellulosic fibres. A process finally described as “dissolution” may therefore include several earlier stages of structural change.

Polyester-containing systems can be interpreted in the same way. Zhang et al. [8] showed that a DES-containing medium combined with an alkaline reagent selectively transformed the PET component in PET/cotton textiles while preserving cotton. Effective PET degradation in such a system requires not only a reactive medium, but also sufficient contact with accessible ester bonds in the polymer matrix. Chemical transformation is therefore coupled to earlier transport and local structural responses, rather than being independent of them [8,44].

This dynamic view also helps explain why similar textile blends may lead to different outcomes. In PET/cotton systems, one study reported selective dissolution of PET with preservation of cotton [15], whereas another achieved the opposite direction of selectivity, removing the cellulosic component while retaining PET fibres [16]. These results should not be read as contradictory evidence for a single mechanism. They reflect different process pathways, determined by the medium, treatment conditions, and accessibility of the individual polymer phases.

In practical terms, DES-assisted separation may begin with medium penetration into the most accessible polymer regions, continue through local reorganization of the polymer structure, and only later develop into more extensive dissolution or degradation. In other cases, this progression may be blocked by high crystallinity, chain orientation, surface barriers, or limited mobility of the medium. Selectivity is therefore better understood as a time-dependent response of the complete DES–polymer system than as a fixed property of either component alone [21,23,31,32,33,34,50,51,52,53].

**Figure 3:**
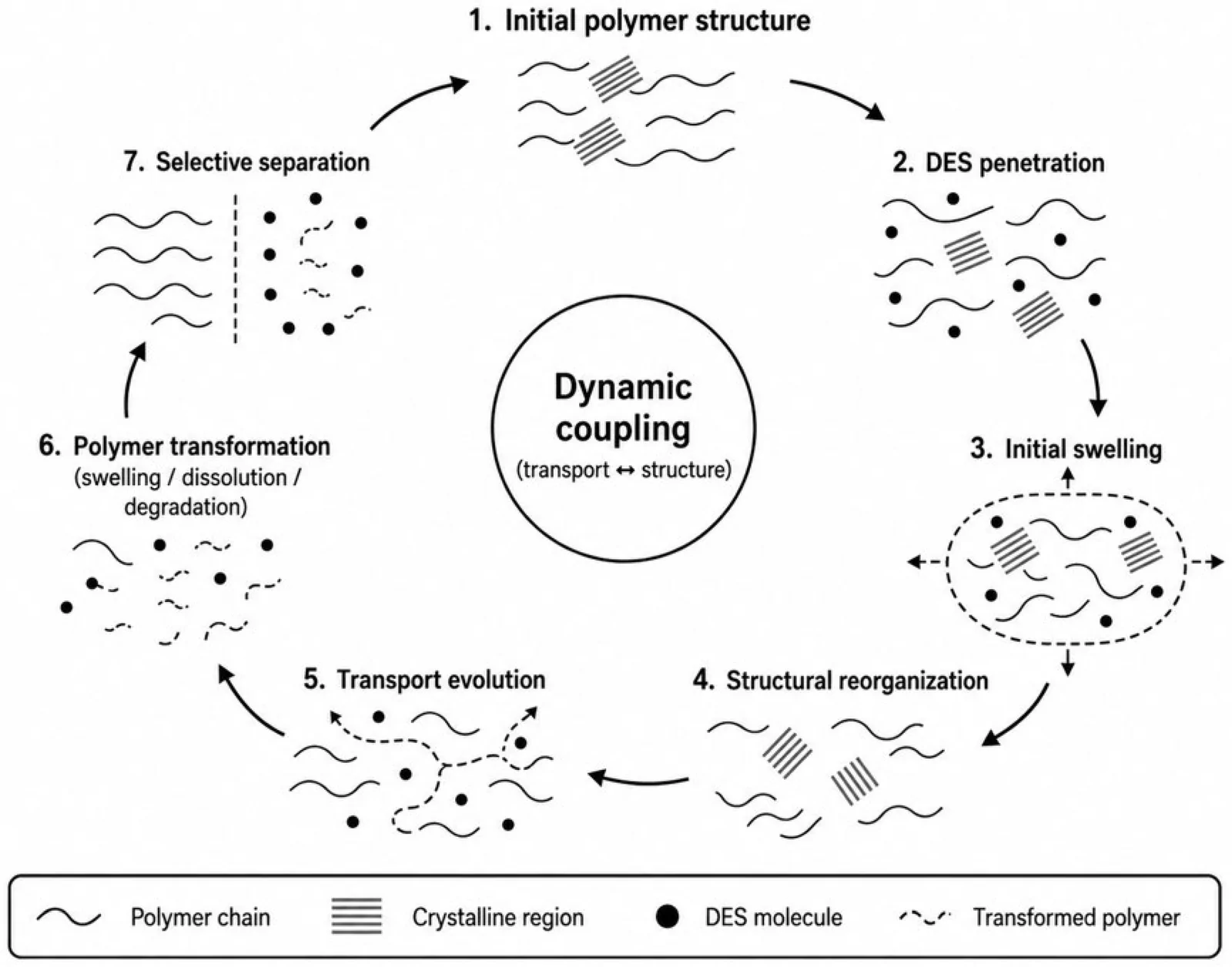
Dynamic mechanistic framework of selective DES–polymer interaction. The scheme illustrates the time-dependent coupling between DES transport and polymer structural evolution, from the initial polymer structure and medium penetration through swelling and structural reorganization to polymer transformation and selective separation.

For this reason, a single final measurement is often insufficient to identify the mechanism of selective separation. More reliable comparison of DES–polymer systems requires monitoring how medium penetration, morphology, crystallinity, molar mass, and chemical structure change during treatment. The dynamic interplay among polymer morphology, solvent transport, and structural evolution is schematically illustrated in Figure 3.

The following subsection therefore summarizes the main polymer-related, DES-related, process-related, and textile-structural factors that may determine which of the above-described developmental scenarios predominates in a given system.

### 3.4. Factors Determining Selective Behaviour

The preceding subsections have shown that the selective behaviour of DES–polymer systems cannot be explained by a single solvent property or by the chemical structure of the polymer alone. The experimentally observed outcome arises from the combined action of several interrelated factors that influence the initial transport of the medium, the extent of supramolecular reorganization, the accessibility of reactive sites, and the subsequent progression of *swelling*, *dissolution*, or *degradation*. Their relative importance may vary substantially among individual systems because it depends on polymer properties, DES composition, process conditions, and the complexity of the textile material itself [21,23,27,31,32,33,34,50,51,52,53].

The first group comprises factors related to polymer structure. Crystallinity, chain orientation, the fraction of amorphous regions, free volume, segmental mobility, and the processing history of the material determine which regions of the polymer matrix are accessible to initial DES penetration. At the same time, the chemical structure of the polymer determines the nature of possible intermolecular interactions, including competition for hydrogen bonding in cellulose and polyamides, coordination with carbonyl groups, or the chemical reactivity of ester, amide, and urethane linkages. Morphological and chemical factors therefore cannot be fully separated: a chemically suitable medium may fail to produce the desired effect when the relevant polymer domains are transport-inaccessible [31,32,33,34,35,50,53,56,57,58,59,60].

The second group includes the properties of the DES itself. The selection of HBA and HBD components, their molar ratio, polarity, viscosity, hydrogen-bonding capacity, acid–base or Lewis-acidic character, and water content determine both the supramolecular organization of the medium and its interaction with the polymer matrix [20,21,22,23,24,25,26,69]. These parameters may act simultaneously, but not necessarily in the same direction. For example, lower viscosity may facilitate handling and medium transport, whereas acidity, basicity, or the presence of coordination-active ions may affect the chemical activation of specific polymer bonds. In DES-catalyzed PET glycolysis, differences in conversion and BHET yield have been associated with the combined effects of acid–base character, viscosity, and DES composition rather than with a single isolated property [44,70,71].

The third group consists of process conditions. Temperature, exposure time, the DES-to-polymer ratio, the ratio of reactive agent to polymer, mixing intensity, particle size, and the mode of energy input may substantially modify both transport rates and chemical transformation. These parameters therefore influence not only the overall efficiency of the process, but also the experimentally observed type of polymer response. In PET glycolysis, for example, changes in temperature, DES amount, and the ethylene glycol-to-PET ratio affected both conversion and selectivity toward BHET [44,70]. Similarly, microwave heating or ultrasonication may alter the rates of energy and mass transfer and thereby affect the time required to achieve a specific degree of modification or degradation [62,72,73].

A distinct group of factors is associated with the properties of real textile materials. Unlike model polymers, post-consumer textiles may contain multiple fibre types, elastane or polyurethane components, dyes, surface finishes, laminates, additives, and degradation-damaged regions. These constituents may act as transport barriers, modify the local accessibility of polymer domains, or affect the purity of the recovered fractions. The resulting selectivity therefore depends not only on the individual interaction between the DES and a particular polymer, but also on its spatial arrangement within the textile architecture and on the presence of other material components [5,6,29,30,35,40,61,66,74].

Overall, no individual parameter can be regarded as a universal predictor of selective behaviour in DES–polymer systems. High chemical affinity, low viscosity, or favourable acid–base activity may promote interaction with a polymer, but their effect will always depend on the transport accessibility of the polymer matrix and the specific process conditions. Selective separation is therefore most accurately understood as the result of the combined influence of polymer-, solvent-, process-related, and textile-structural factors. Their systematic classification is summarized in Table 6.

## 4. Mechanistic Behaviour of Individual Textile Polymers

The mechanistic framework introduced in Section 3 provides a common basis for interpreting interactions between DES and individual textile polymers. However, this does not imply that all polymers interact with DES through the same pathway or that their selective behaviour can be inferred solely from the chemical structure of the repeating unit. The resulting response always depends on the combined effects of chemical functional groups, supramolecular organization, morphology, transport accessibility, and specific process conditions [20,21,22,23,24,25,26,31,32,33,34,50,51,52,53].

For cellulosic fibres, the dense network of intra- and intermolecular hydrogen bonds, and its disruption or preservation during contact with the medium, are of primary importance. In polyester materials, high crystallinity and fibre orientation are particularly relevant, together with the accessibility of ester bonds to hydrolysis or glycolysis. Polyamides combine strong hydrogen bonding between amide groups with sensitivity to chemical and hydrolytic environments, whereas, in polyurethanes, segmental morphology and the different stability of soft and hard segments are important, along with the possibility of urethane-bond cleavage. In real textiles, these material-specific features are further complicated by the spatial arrangement of fibres, the presence of additional polymers, dyes, surface finishes, and degradation-altered regions [5,6,29,30,35,40,61,74].

The following subsections are therefore not intended as a simple overview of “suitable DES” for individual polymers. Their aim is to compare which mechanistic factors are most relevant in specific material systems, which experimental responses have actually been observed in the literature, and to what extent these findings can be interpreted in terms of transport, swelling, supramolecular reorganization, dissolution, or chemical degradation. This approach also makes it possible to distinguish directly supported experimental conclusions from cautious mechanistic interpretation and to compare different DES–polymer systems more consistently [8,14,15,16,21,23,27,28].

Given the uneven availability of direct mechanistic evidence across textile polymer classes, the following discussion focuses on fibre systems for which DES-related experimental evidence is currently most informative. Protein-based fibres are considered briefly as an illustrative case within multicomponent textile systems.

### 4.1. Cellulose-Based Fibres

Cellulosic fibres represent a distinct group of textile polymers from a mechanistic perspective because their behaviour in DES systems is strongly influenced by the dense network of intra- and intermolecular hydrogen bonds, semicrystalline morphology, and hierarchical fibrillar organization. The hydroxyl groups of cellulose provide a large number of potential interaction sites; however, their accessibility is limited by the arrangement of polymer chains into crystalline domains and by the stability of the original supramolecular network. The interaction between a DES and cellulose therefore does not generally begin with immediate molecular dissolution, but rather with penetration of the medium into more accessible regions, gradual disruption of hydrogen bonds, and changes in the local organization of the fibrillar structure [55,67,68,75,76,77].

A fundamental requirement for effective interaction is the ability of the DES to compete with the original hydrogen bonds in cellulose while simultaneously providing sufficient transport accessibility of the medium. The mere presence of components capable of hydrogen bonding therefore does not automatically imply high dissolution capacity. The molar ratio of the components, system viscosity, temperature, water content, and the ability of the DES constituents to establish new interactions with the hydroxyl groups of cellulose are also critical. The resulting response may range from surface swelling and decrystallization to recoverable dissolution or acid-assisted degradation [17,18,55,67,68,76]. Evidence from related ionic-liquid systems further indicates that changes in crystallinity or increased accessibility of the cellulosic fraction do not necessarily imply direct molecular dissolution of cellulose itself. During the pretreatment of lignocellulosic biomass, systems have been reported in which improved subsequent processability was primarily associated with the selective removal of lignin components, without substantial decrystallization of cellulose [78]. Although such material systems are not directly comparable to cotton fibres, they emphasize the need for cautious interpretation of changes in crystallinity and morphology in complex cellulosic materials.

The different behaviour of choline chloride-based DES was documented by Ren et al. [68], who compared several systems containing different hydrogen-bond donors. The ChCl/imidazole system exhibited the highest dissolution capacity toward cellulose, while the addition of polyethylene glycol further increased the accessibility of the medium to the cellulosic structure. The authors observed a decrease in the crystallinity of regenerated cellulose and did not identify the formation of new derivatives, which is consistent with an interpretation of the process as direct dissolution accompanied by structural reorganization. This example shows that DES performance is governed not only by hydrogen-bonding ability, but also by viscosity and the accessibility of interaction sites within the solvent medium [68].

A similar trend was confirmed by Zhang et al. [67], who compared ChCl-based DES containing oxalic acid, citric acid, urea, and glycerol. Under the investigated conditions, the cellulose dissolution capacity followed the order oxalic acid/ChCl > citric acid/ChCl > urea/ChCl > glycerol/ChCl. The higher efficiency of the acidic systems was attributed to a more favourable combination of hydrogen-bonding interactions, lower viscosity, and higher mobility of the medium components. In the oxalic acid/ChCl system, regeneration resulted in a change in crystalline form from cellulose I to cellulose II, while the chemical structure of the regenerated phase remained comparable to that of the original cellulose according to FTIR analysis [67]. This result emphasizes that a reduction in crystallinity or a change in crystalline modification does not, by itself, indicate degradation of the polymer chains.

More recently, Zhao et al. reported that a ternary ChOH/urea/zinc glycinate DES containing 3.85 wt% water dissolved 12 wt% microcrystalline cellulose with a degree of polymerization of approximately 150 at 65 °C within 60 min [49]. The system retained more than 80% of its cellulose-solubilization efficiency after three reuse cycles. These results provide quantitative evidence that cellulose dissolution performance should be evaluated not only by structural changes in the regenerated polymer, but also by the attainable polymer concentration and solvent reusability. However, because the study used microcrystalline cellulose rather than textile fibres, the transferability of the reported performance to highly oriented cotton structures remains to be demonstrated.

An important alternative scenario is represented by DES pretreatment of cotton, in which the objective is not complete cellulose dissolution but rather its increased accessibility for subsequent transformation. Liu et al. [55] used a ChCl/oxalic acid·2H_2_O system in combination with microwave heating and ultrasonication to prepare cellulose nanocrystals from cotton. A 3 min microwave pretreatment at 80 °C followed by ultrasonic fibrillation yielded cellulose nanocrystals with a yield of 74.2%. In this case, the DES promoted disruption of strong hydrogen bonds and increased the accessibility of the fibre structure; the resulting process therefore cannot be interpreted as simple molecular dissolution of cellulose [55].

In real polyester/cotton textiles, the mechanisms become even more differentiated. Wang et al. [79] used a ChCl/TsOH DES for the selective processing of polyester/cotton textiles. Under optimized conditions of 75 vol.% DES, 110 °C, and 10 min, the cotton component was degraded while PET remained preserved. The authors obtained 99.20% recycled PET, 69.46% microcrystalline cellulose, and 38.91% glucose. This system therefore does not represent merely selective dissolution of cellulose, but rather a combination of structural destabilization and partial chemical degradation, the extent of which depends on the acidity of the medium and the process conditions [79].

A different outcome was obtained in a NADES system based on ChCl/lactic acid for the treatment of waste PET/cotton textiles. Ara et al. [80] isolated cotton and polyester fractions with high purity after exposure at 110 °C for 3 h; the cotton yield reached approximately 98%, while the polyester yield was 99%. According to the authors, the recovered PET remained undamaged, whereas the cellulosic fraction exhibited partial degradation. This example shows that, for a similar type of textile blend, a DES system may enable selective fractionation without complete degradation of both polymer components, with the outcome depending on the specific composition of the medium and the treatment intensity [80].

In a more complex PET/cotton/PU system, Zeng et al. [66] used a ZnCl_2_/AlCl_3_/H_2_O medium for the targeted dissolution of the cotton component at room temperature. Polyester and polyurethane fibres remained in the solid phase after cellulose removal and were subsequently separated by additional steps. The authors interpreted the selectivity in terms of the action of metal ions and chloride anions, which disrupt the hydrogen-bonding network of cellulose and promote its solubilization. From the perspective of this review, the key point is that the selective interaction with cellulose was not determined solely by the presence of hydroxyl groups, but by the combined effects of the chemical character of the medium, the accessibility of the fibre structure, and transport conditions in a real multicomponent textile [66].

These examples demonstrate that the behaviour of cellulose in DES systems cannot be reduced to the question of whether the material “dissolves” or “does not dissolve.” Depending on the DES formulation and process conditions, the predominant response may involve swelling, decrystallization, recoverable dissolution, nanocellulose formation, selective removal of cotton from a blended textile, or partial hydrolytic degradation. Mechanistic interpretation therefore requires distinguishing whether concurrent changes in crystallinity, molar mass, chemical structure, and fibre-phase morphology have been demonstrated [55,66,67,68,76,79]. Representative DES-based approaches reported for cellulose-based fibres and cotton-containing textile systems are summarized in Table 7.

### 4.2. Polyester Fibres

Polyester fibres, represented predominantly by poly(ethylene terephthalate) (PET), constitute one of the most important groups of synthetic textile polymers. Their behaviour in DES systems differs mechanistically from that of cellulose because the dominant factors are not an extensive hydrogen-bonding network, but rather the combined effects of ester-bond reactivity, crystallinity, chain orientation, and transport accessibility of amorphous regions. PET fibres are typically highly oriented and semicrystalline, with crystalline domains potentially limiting the penetration of the processing medium toward ester linkages. Consequently, chemical affinity of a DES or reactive agent toward ester groups does not necessarily result in uniform degradation or dissolution of the entire polymer phase [31,32,33,34,35,50,53,56,57,58,59,60].

Three principal types of DES-assisted interaction with PET can be distinguished in the literature. The first group comprises systems in which DES acts primarily as a catalytically active medium during PET glycolysis or hydrolytic degradation. The second group includes systems promoting surface activation or partial hydrolysis of PET fibres without complete depolymerization of the material. The third group consists of solvent systems enabling the selective dissolution of PET from blended textiles while preserving the cellulosic component. These approaches yield different products and therefore cannot be interpreted as the same mechanism merely because all involve contact between PET and a DES-based medium [8,14,15,44,62,70,71,73,81,82,83].

The importance of DES chemical composition in PET glycolysis was demonstrated by Sert et al. [44], who compared several eutectic systems under the same basic conditions. The K_2_CO_3_:ethylene glycol system exhibited the highest efficiency, whereas ChCl:glycerol did not lead to BHET formation. Under optimized conditions, including a temperature of 180 °C, a higher ethylene glycol-to-PET ratio, and an increased amount of DES, complete PET conversion and an 88% BHET yield were achieved. These results indicate that PET glycolysis requires simultaneous consideration of basicity, viscosity, the amount of reaction medium, and temperature. The name or chemical class of the DES alone is therefore insufficient to explain the observed performance [44].

Similarly, pronounced differences among DES systems containing different metal salts were observed for post-consumer PET. Ha et al. [70] compared choline chloride- or urea-based systems combined with zinc, manganese, and cobalt salts. The best results were obtained using ChCl:Zn(OAc)_2_, for which PET conversion above 99% and BHET selectivity of 91.6% were achieved after optimization of the process conditions. The authors associated the high activity of the system with the combined contribution of hydrogen-bonding and coordination interactions among the DES components, ethylene glycol, and the carbonyl groups of PET. Although this mechanism is supported by a combination of experimental data and computational modelling, it remains important from the perspective of textile materials that the resulting efficiency also depends on the accessibility of ester bonds within the specific polymer morphology [70].

More recently, Ricci et al. reported rapid PET depolymerization using a transition-metal-free KF:EG (1:6) DES. Under anhydrous conditions, quantitative PET conversion was achieved at 180 °C within 15 min, while microwave irradiation at 90 W reduced the reaction time to 3 min; quantitative PET consumption and monomer recovery were maintained over five consecutive heat-induced cycles [84]. A second-order kinetic analysis yielded an apparent activation energy of 156.6 kJ mol^−1^. Controlled hydration shifted product selectivity toward terephthalic acid, reaching up to 97%, but caused fluoride losses of up to 85%, attributed to HF formation. This system therefore demonstrates that water content may simultaneously alter the reaction pathway, product selectivity, catalyst stability, and process safety.

The practical relevance of DES-catalyzed glycolysis for blended textiles was demonstrated by Liu et al. [14] using a betaine:Zn(OAc)_2_ system. For neat PET, complete conversion and an 87.2% BHET yield were achieved. For PET/cotton textiles, the same system resulted in complete degradation of the polyester component, an approximately 85% BHET yield, and approximately 95% recovery of cotton. This result is important because it shows that selective glycolysis can be used not only for PET depolymerization but also for preservation of the second polymer fraction. At the same time, the system operates at 190 °C and therefore represents chemical transformation of PET rather than simple polyester dissolution [14].

A different route is represented by DES-assisted alkaline hydrolysis of PET. Zhang et al. [8] used a ChCl:ethylene glycol system in combination with NaOH to process PET and PET/cotton textiles. For the blended textile, complete PET degradation, a terephthalic acid yield of 98.95%, and cotton mass loss below 3% were achieved under relatively mild conditions of 98 °C and 60 min. The authors interpreted the role of the DES as activation of the PET surface and facilitation of subsequent ester-bond cleavage in an alkaline environment. This example emphasizes that the DES may function as part of a reactive medium promoting hydrolysis, while the resulting process should be classified as chemical degradation rather than molecular dissolution of PET [8].

Surface treatment of PET may also be accompanied by significant changes in properties without complete polymer depolymerization. During microwave treatment of PET textiles in ChCl:ethylene glycol or ChCl:urea systems containing low amounts of NaOH, a pronounced increase in wettability and immediate water wicking were observed, while the mechanical properties of the fibres were affected to different extents [81,82]. Such results primarily indicate surface activation or partial hydrolysis rather than evidence of complete PET degradation. Their relevance to this review lies in demonstrating the sensitivity of PET fibres to the combined effects of DES composition, alkaline agent, microwave heating, and exposure time.

Very rapid PET depolymerization from blended textiles was reported by Choi and Choi [62] using a ChCl:glycerol/NaOH system under microwave heating. For different PET/cotton textiles, the PET component was reported to be completely depolymerized within 80–100 s. This finding highlights the strong influence of the combined chemical environment and mode of energy transfer. However, the high process rates alone do not allow the individual contributions of microwave heating, alkaline hydrolysis, medium transport, and a possible catalytic effect of the DES to be distinguished without more detailed time-resolved investigation [62].

A distinct group of systems aims to preserve PET as a polymer. Depope et al. [15] used a hydrophobic menthol:benzoic acid DES to treat PET/cotton textiles and reported complete dissolution of PET while retaining cotton. Following regeneration, 97% of PET and 100% of the cotton fraction were recovered. In contrast to glycolysis or alkaline hydrolysis, this approach aims at separation of polymer fractions without intentional PET depolymerization. It therefore provides an important example of how the same polyester/cotton material can be processed through entirely different pathways depending on the DES type and process conditions [15].

From a mechanistic perspective, the behaviour of PET in DES systems cannot therefore be reduced to the simple category of “polyester dissolution.” Depending on the composition of the medium and process conditions, PET may undergo only surface activation, selective alkaline hydrolysis, glycolytic depolymerization, or selective dissolution while preserving polymer chains. Distinguishing among these outcomes is particularly important in textile recycling because it determines whether the final product is regenerated PET polymer, BHET, TPA, or merely a modified fibre. The main DES-assisted approaches for PET and PET-containing textiles are summarized in Table 8.

### 4.3. Polyamide Fibres

Polyamide fibres, particularly polyamide 6 (PA6) and polyamide 6,6 (PA66), differ from both cellulose and PET in their high density of amide groups capable of forming strong intermolecular hydrogen bonds. These interactions, together with crystallinity, chain orientation, and the processing history of the fibre, strongly influence fibre strength, transport accessibility, and response to liquid media. The behaviour of nylon fibres in DES systems therefore cannot be explained solely by the chemical reactivity of the amide bond; the ability of the medium to penetrate amorphous regions and disrupt the existing supramolecular organization is also important [85,86,87].

Water content is another relevant factor. Compared with PET, polyamides are more susceptible to water sorption, with water preferentially penetrating amorphous regions, interacting with amide groups, and increasing chain segmental mobility. In DES systems, water should therefore not be regarded merely as a component that reduces medium viscosity. It may simultaneously alter hydrogen-bonding interactions within the DES, plasticize the polymer matrix, and affect both medium transport and the subsequent hydrolytic stability of amide bonds [87].

From a mechanistic perspective, two basic interaction regimes can be distinguished for polyamides. The first involves medium sorption, swelling, softening, or partial reorganization of the hydrogen-bonding network without clearly demonstrated chain scission. The second involves chemical depolymerization, in which hydrolysis or other cleavage of amide bonds leads to the formation of low-molar-mass products. Distinguishing between these regimes is important because a visual change in the fibre or loss of mechanical integrity alone does not prove chemical degradation of the polyamide.

The most convincing direct DES example was provided by Rollo et al. [88], who investigated the hydrolytic depolymerization of PA66 using ferric Lewis/Brønsted-acidic DES. Using FeCl_3_·6H_2_O:acetic acid and FeCl_3_·6H_2_O:methanesulfonic acid systems, quantitative PA66 conversion was achieved at 180 °C after 5 h. Following optimization of the reaction and product-isolation procedures, the authors obtained adipic acid and hexamethylenediamine in the form of a diammonium salt, with an overall monomer yield exceeding 85%. The process was demonstrated not only for PA66 pellets, but also for PA66 fibres and real post-consumer nylon hosiery textiles [88]. This result clearly shows that, in a sufficiently acidic and reactive DES system, the interaction may progress beyond swelling or simple dissolution and lead to complete chemical depolymerization of amide bonds.

A different and milder example is provided by the treatment of PA6 fishing nets in a choline chloride:monoethanolamine (ChCl:MEA) system. Castillo et al. [89] evaluated the effects of DES composition, the amount of 4-(dimethylamino)pyridine, the net-to-DES ratio, and process parameters. Under optimized conditions of 156 °C and 2 h, approximately 80% PA6 conversion was achieved. The degradation process was monitored by ATR-FTIR through changes associated with chain cleavage [89]. Because this study was reported as a brief conference contribution without detailed quantification of all products, the findings should be interpreted cautiously as evidence of promising DES-assisted PA6 solvolysis rather than as a fully established monomer-recycling process.

In textile blends, polyamide does not always act as the target phase for depolymerization. It may instead be the preserved component when the processing medium selectively destabilizes another polymer. Johansen et al. [90] showed that solvolysis of the elastane component in tert-amyl alcohol containing a catalytic amount of KOH enabled the isolation of a residual polyamide textile from nylon/elastane blends. FTIR and DSC analyses indicated elastane removal while preserving the polyamide phase. Although this system is not DES-based, it provides an important reference example of the selective preservation of polyamide in a real textile blend and also provides a natural transition to the following subsection on polyurethane and elastane [90].

The current literature therefore indicates that polyamides may remain relatively stable textile phases or undergo complete chemical depolymerization, depending on the medium and process conditions. In contrast to PET and cellulose, however, comprehensive systematic comparisons of PA6, PA66, and real nylon textiles under comparable DES conditions are still lacking. Studies that simultaneously monitor swelling, fibre morphology, crystallinity, molar mass, and the composition of degradation products are particularly needed. Without such combined characterization, it is not possible to reliably distinguish whether a DES causes only structural destabilization of a polyamide or genuine cleavage of amide-containing polymer chains. Representative DES-related and benchmark approaches reported for polyamide systems are summarized in Table 9.

### 4.4. Polyurethane and Elastane Systems

Polyurethanes and elastane fibres represent a particularly complex class of materials from the perspective of selective recycling. Unlike cellulose, PET, or polyamides, their behaviour is governed not only by the chemical structure of the repeating unit, but also by their segmented morphology. Thermoplastic polyurethanes and many elastane materials contain relatively flexible soft segments and more rigid hard segments stabilized by the association of urethane or urea groups. The extent of microphase separation, hydrogen-bond density, and the nature of the soft segments may therefore strongly influence medium transport, the local accessibility of bonds, and the subsequent chemical response of the material [74,91,92].

In DES–polyurethane systems, it is necessary to distinguish between physical penetration of the medium into the polymer matrix, dissolution or dispersion of polymer fragments, and genuine chemical depolymerization. Available primary studies indicate that, in sufficiently reactive DES systems, cleavage of urethane, carbamate, or urea linkages may occur, accompanied by a decrease in molar mass and the formation of low-molar-mass products. The disappearance of the original polymer form should therefore not automatically be interpreted as molecular dissolution in the absence of chemical transformation.

Zhang et al. [93] demonstrated the controlled degradation of a polycarbonate polyurethane elastomer in a ChCl:urea (1:2) system. Using a combination of FTIR, NMR, and MALDI-TOF-MS, the authors demonstrated selective cleavage of carbamate and urea linkages with relatively limited damage to carbonate bonds. At 170 °C and a reaction time of 8 h, complete PU degradation and a 57.4% yield of polycarbonate diol were achieved. This example is particularly important because it demonstrates the possibility of controlled chemical transformation of segmented PU while preserving the more valuable polycarbonate fraction [93].

The role of transport and process kinetics is suggested by the study of Mohd Razib et al. [72], who used a ChCl:urea DES for the degradation of thermoplastic polyurethane with ultrasonic assistance. At 150 °C, ultrasonication increased the determined degree of degradation from 58.51 ± 0.04% to 63.71 ± 0.03%. GPC confirmed a pronounced decrease in molar mass, while FTIR and NMR provided evidence for the formation of low-molar-mass products, including *o*-toluidine. The authors attributed the enhanced effect to cavitation phenomena, which likely promoted mass transfer and accelerated polymer-chain cleavage [72]. This result supports the interpretation that, in PU systems, DES efficiency may depend not only on the chemical composition of the medium, but also on the manner in which the medium accesses the microphase-separated polymer structure.

Kaur et al. [65] reported combined dissolution and depolymerization of PU in a lactic acid:ZnCl_2_-based DES. At a molar ratio of 1:1, a temperature of 60 °C, and under white LED irradiation, approximately 70 wt.% PU dissolution was achieved. The regenerated material exhibited a lower molar mass, approximately 900–3000 g·mol^−1^, and several analytical methods confirmed cleavage of urethane bonds together with the formation of new functional groups [65]. This system should therefore not be classified as simple PU dissolution; the experimental evidence supports interpretation as a combined process in which dissolution is accompanied by chemical depolymerization. A broader review of PU/DES systems likewise identifies DES composition, temperature, and processing time as major parameters influencing the outcome of polyurethane solubilization or degradation [94].

For elastane fibres, direct DES-based evidence has only recently begun to emerge. Although elastane is structurally classified as a segmented polyurethane or polyurethane–urea elastomer, its behaviour may be affected by high fibre orientation, very small diameter, the presence of textile surface finishes, and its spatial arrangement within blended yarns. Results obtained for model PU elastomers or thermoplastic polyurethanes therefore cannot be directly transferred to elastane in real textiles.

Depope et al. demonstrated the selective dissolution of elastane from PET/EL, PA6/EL, and PA66/EL textiles using DL-menthol:benzoic acid (MeBA, 3:1) and DL-menthol:lauric acid (MeLA, 2:1) DES systems [95]. Complete elastane removal was achieved within 10–20 min at 140–170 °C, depending on the matrix polymer and DES formulation, while SEM, ATR-FTIR, DSC, TGA, and mechanical testing indicated preservation of the PET, PA6, and PA66 fibres. Both DES systems maintained near-quantitative elastane-removal efficiency over five reuse cycles, and the recovered matrix polymers were successfully re-extruded into continuous filaments. Mechanistically, the results are consistent with selective disruption and dissolution of the hydrogen-bonded elastane phase rather than degradation of the companion fibres, although the optimum process window remained dependent on the textile blend.

This distinction is clearly illustrated by the multistep treatment of PET/cotton/PU textiles reported by Zeng et al. [66]. In the first step, the cotton component was selectively removed using a ZnCl_2_/AlCl_3_/H_2_O DES, whereas PET and PU remained in the solid phase. Polyurethane was subsequently separated from PET using DMSO. This study therefore does not provide evidence for DES-induced PU dissolution, but it demonstrates that the relative stability of PU toward a particular DES may be purposefully exploited in a sequential separation strategy for multicomponent textiles [66].

The available studies therefore support the view that the resulting interaction between polyurethanes and DES may depend strongly on segmented morphology, urethane-bond reactivity, soft-segment type, the presence of Lewis-acidic or hydrogen-bonding components, and process conditions. Despite this progress, systematic comparisons of model PU, TPU, and elastane fibres under identical DES formulations remain limited. Future work should therefore simultaneously monitor medium transport, changes in microphase morphology, molar mass, chemical bonds, and the nature of the resulting products in order to reliably distinguish between swelling, dissolution, and depolymerization. Representative DES-based approaches reported for polyurethane and elastane systems are summarized in Table 10.

### 4.5. Mechanistic Interpretation of Multicomponent Textile Systems

The preceding subsections have shown that the behaviour of individual polymers in DES systems is governed by different combinations of chemical structure, supramolecular organization, transport accessibility, and process conditions. In real textile waste, however, these polymers do not occur in isolation, but are incorporated into blended yarns, fabrics, or multilayer materials containing natural and synthetic fibres, elastane components, dyes, surface finishes, and other additives. Selective separation therefore cannot be evaluated solely on the basis of the behaviour of a single neat polymer, but must be assessed in relation to the entire material system and to the preservation or transformation of all relevant fractions [5,6,29,30,35,40,61].

The largest number of examples is available for PET/cotton textiles. For comparable polyester/cotton blends, fundamentally different strategies have been reported, leading to different target products. Depope et al. [15] used a hydrophobic menthol:benzoic acid DES for the selective dissolution of PET while preserving the cotton component. According to the authors, PET was completely dissolved within several minutes and subsequently regenerated with a yield of 97%, whereas cotton was recovered with complete material retention. This approach is directed toward the separation of polymer fractions while preserving PET as a macromolecular material.

A different route involves targeted chemical transformation of the polyester component. Liu et al. [14] used betaine:Zn(OAc)_2_ for the glycolysis of PET/cotton textiles, in which PET was depolymerized to BHET while most of the cotton component remained preserved. Zhang et al. [8] achieved selective alkaline degradation of PET with minimal cotton loss in a ChCl:ethylene glycol/NaOH system, whereas Choi and Choi [62] used a combination of ChCl:glycerol, NaOH, and microwave heating for rapid PET removal from polyester/cotton textiles. These processes cannot be interpreted as selective PET dissolution because they involve cleavage of ester bonds and the formation of low-molar-mass products or monomers.

The opposite direction of selectivity has been reported for systems targeting the cellulosic phase. Wang et al. [79] used ChCl:TsOH for degradation of the cotton component in PET/cotton textiles while preserving the polyester fraction. Yang et al. [16] used a ZnCl_2_/H_3_PO_4_/H_2_O system for selective dissolution of cellulose while retaining PET fibres. Although these studies employed different media and process conditions, they collectively demonstrate that, for the same basic type of textile blend, either PET or cotton may serve as the target phase.

In more complex textile systems, the importance of sequential separation becomes even greater. Zeng et al. [66] processed PET/cotton/PU textiles through several consecutive steps. In the first step, a ZnCl_2_/AlCl_3_/H_2_O DES selectively dissolved cotton at room temperature, while PET and PU remained in the solid phase. Polyurethane was subsequently separated using DMSO, and the polyester component was dissolved and depolymerized in a separate subsequent step. This example is particularly important because it demonstrates that, in multicomponent textiles, a single solvent system may not provide complete separation of all components. A successful strategy may instead consist of several selective steps, each targeting a different polymer phase.

Recent evidence extends this concept to fully synthetic elastane-containing textiles. Depope et al. selectively removed elastane from PET/EL, PA6/EL, and PA66/EL blends using menthol-based DES while preserving the main polymer fibres and enabling their subsequent re-extrusion [95]. The blend-dependent temperature–time windows demonstrate that selectivity toward the same target component may still depend strongly on the companion polymer and textile architecture.

Although current literature has focused predominantly on polyester/cotton blends, protein-based textiles represent another group in which a clear distinction must be made between dissolution, structural destabilization, and chemical transformation. Pääkkönen et al. [96] observed a sequential interaction during the treatment of waste wool using a ChCl:lactic acid system, in which the DES first removed cuticular scales from the fibre surface and subsequently degraded the cortical layer. The resulting water-soluble keratin retained characteristic amide groups but exhibited altered secondary structure and low molar mass. This example demonstrates that, even for protein fibres, the designation “dissolution” does not necessarily imply preservation of the original polymer structure, but may involve sequential surface destabilization and partial material degradation [96].

These examples cannot be directly compared as evidence of a single universal mechanism because they differ in the media used, temperature, reaction time, mode of energy transfer, and textile architecture. They nevertheless demonstrate that the resulting selectivity is not a stable property of PET, cotton, or a DES formulation alone. Rather, it is the outcome of a specific process configuration combining chemical interactions, transport accessibility of polymer domains, fibre morphology, and processing conditions.

The mechanistic framework proposed in Section 3 is therefore not intended to predict the outcome of every new system, but to provide a common basis for comparing reported approaches. It enables distinction between processes aimed at preserving a polymer fraction, molecular dissolution, partial structural destabilization, or chemical depolymerization. For real blended textiles, such differentiation is essential because the same designation of “separation” may correspond to fundamentally different material outcomes across different studies. Representative selective separation strategies reported for multicomponent textile systems are summarized in Table 11.

## 5. Unified Mechanistic Interpretation and Practical Implications of DES-Assisted Textile Recycling

The previous chapters show that the selective behaviour of deep eutectic solvents in textile recycling cannot be explained simply by chemical affinity between the solvent and the polymer. Chemical structure is clearly important, but it is only part of the picture. Across the available studies, the outcome also depends strongly on how the medium enters the polymer, which regions of the material are accessible, how the polymer morphology responds, and how the structure evolves during contact with the DES [5,6,20,21,24,28,29,30,31,32,33,34,35,37,38,40,41,50,51,52,53,56,57,58,59,60,61,69,75,76,77,90,93,97,98,99].

When the results for different textile polymers are considered together, a common logic begins to emerge. Cellulose, PET, polyamides, and polyurethanes differ substantially in chemistry and morphology, but the same basic questions are relevant in each case: where can the medium penetrate, which intermolecular interactions are disturbed, does the material only swell or does it dissolve, and is there evidence of chemical transformation?

The literature does not point to one universal mechanism for DES-assisted textile recycling. It does, however, reveal recurring mechanistic levels that help compare different systems. These include the accessibility of polymer domains to the medium, the reorganization of intermolecular interactions, the possible progression from swelling to dissolution or chemical transformation, and the role of processing conditions in determining the final material product. In cellulose, the key issues are hydrogen bonding and fibrillar morphology; in PET, crystallinity, chain orientation, and ester-bond reactivity are central; in polyamides, the stability of amide bonds must be considered; and in polyurethanes, segmented morphology and the chemical sensitivity of urethane linkages become particularly important [44,55,62,65,67,68,70,71,72,81,82,83,88,89].

This shared way of reading the evidence also explains why similar textile blends may end up following different recycling routes. A PET/cotton material, for example, can be treated in a way that dissolves and regenerates PET, depolymerizes PET through glycolysis or hydrolysis, or removes the cellulosic fraction while preserving polyester. These outcomes are not necessarily contradictory. They reflect different process pathways created by different combinations of medium composition, material morphology, accessibility, and processing conditions [8,14,15,62,66,79].

A unified mechanistic interpretation therefore does not mean that all DES–polymer systems behave in the same way. Rather, it provides a common set of questions for evaluating them: which polymer domains are actually accessible to the medium, which interactions are changed, whether polymer chains are preserved or cleaved, and what type of material product is ultimately obtained. Future studies could strengthen this interpretation by combining experimental characterization with molecular modelling, particularly for understanding local DES–polymer interactions and the supramolecular organization of DES media [100].

Because the available studies differ substantially in material type, DES composition, additives, and characterization depth, the proposed framework should be understood as a comparative interpretive tool. It distinguishes evidence-supported mechanisms from plausible mechanistic interpretations, but it is not intended to rank solvent systems quantitatively or to predict selectivity without experimental validation. Representative experimental observations supporting this common mechanistic interpretation are summarized in Table 12.

Although the framework is not intended to provide a universal quantitative ranking, the available evidence nevertheless identifies several systems that merit priority for further validation. ChOH/urea/zinc glycinate combines a relatively high cellulose loading with partial retention of dissolution performance over repeated cycles [49]. KF:EG enables rapid and reusable PET depolymerization, although hydration-dependent fluoride loss and possible HF formation require careful control [84]. Menthol-based MeBA and MeLA systems appear particularly promising for elastane-containing synthetic textiles because they enabled selective elastane removal from PET- and PA-based blends while preserving the matrix polymers and allowing their subsequent re-extrusion [95]. ChCl/lactic acid and ZnCl_2_/AlCl_3_/H_2_O also merit further investigation for multicomponent textile fractionation, although their broader applicability and long-term solvent stability remain to be established [66,80].

As the field develops, additional effective DES formulations and processing strategies are likely to emerge. Their meaningful assessment will, however, require more than demonstrating high conversion or apparent component removal. The mechanistic framework outlined in this review may provide a useful basis for evaluating such systems by considering polymer morphology, transport accessibility, structural evolution, chemical transformation, textile architecture, and the intended recovery pathway together.

### 5.1. Practical Implementation and Scale-Up

Demonstration of selective dissolution or depolymerization at laboratory scale does not by itself establish the practical feasibility of a DES-based textile-recycling process. Translation toward larger-scale operation requires evaluation of the complete processing sequence, including textile impregnation, heat and mass transfer, separation of solid and dissolved fractions, recovery and purification of the target products, regeneration of the DES, and management of water, antisolvents, additives, and accumulated contaminants. These requirements are particularly important for viscous DES systems and heterogeneous textile feedstocks, in which incomplete penetration, inefficient mixing, and retention of the processing medium within the fibre structure may substantially increase solvent consumption, processing time, and energy demand.

Solvent regeneration should be assessed not only by the number of reported reuse cycles, but also by DES recovery yield, preservation of its composition and water content, retained process efficiency, and the accumulation of dissolved polymers, degradation products, dyes, finishes, and other impurities. Depending on the process, regeneration may include filtration of retained solids, precipitation and recovery of the dissolved polymer using an antisolvent, removal of water or volatile antisolvent, and restoration of the original DES composition before reuse. Recent studies have reported partial or near-quantitative retention of performance over three to five cycles in cellulose dissolution, elastane removal, and PET depolymerization systems [49,84,95]. These results are encouraging, but they were obtained using defined feedstocks and relatively limited cycle numbers. Validation using real post-consumer textiles should therefore include complete solvent and product mass balances together with monitoring of viscosity, chemical composition, selectivity, and recovered-product purity.

The energy and safety requirements of the complete process must also be considered. Energy demand arises not only from the nominal treatment temperature, but also from heating and mixing viscous media, microwave or ultrasonic operation, cooling, antisolvent recovery, product drying, and DES regeneration. Scale-up may further alter heat and mass transfer, particularly in densely packed, coated, or highly oriented textile structures. Systems containing strong acids or bases, metal salts, or fluoride components additionally require assessment of equipment corrosion, worker exposure, and waste-stream management. In the hydrated KF:EG system, for example, fluoride loss attributed to HF formation demonstrates how changes in solvent composition may introduce additional safety limitations [84]. Further development should therefore combine pilot-scale validation with solvent-recovery studies, complete mass balances, techno-economic analysis, and life-cycle assessment, using established solvent-based and chemical-recycling routes as appropriate benchmarks.

### 5.2. Future Research Priorities

Future studies should improve the comparability of DES–polymer systems through consistent reporting of DES composition and water content, polymer-to-solvent ratio, polymer morphology, textile architecture, and treatment conditions. Time-resolved characterization combining solvent uptake, microscopy, crystallinity, molar mass, chemical structure, and product analysis is particularly needed to distinguish swelling, dissolution, and chemical degradation and to identify situations in which several pathways coexist [55,66,67,68,88,89].

Promising systems should subsequently be validated using real post-consumer and multicomponent textiles, where dyes, finishes, fibre orientation, and contaminants may substantially alter transport and selectivity [35,66,95]. Reuse studies should extend beyond a limited number of cycles and assess changes in DES composition, process efficiency, selectivity, and recovered-product quality [49,84,95]. Molecular modelling may support the interpretation of local DES–polymer interactions [100], while data-driven and inverse-design approaches could assist the future screening of solvent formulations [101]. Process-level requirements concerning solvent recovery, safety, environmental performance, and scale-up are discussed in Section 5.1.

## 6. Conclusions

This review shows that the central question in DES-assisted textile recycling is not simply whether a particular medium can dissolve or degrade a polymer, but what material outcome it produces and which polymer fractions retain their value. Depending on the polymer, DES formulation, and processing conditions, the desired pathway may involve swelling, structural destabilization, selective dissolution and regeneration, or controlled depolymerization into recoverable low-molar-mass products.

Evidence from cellulose, PET, polyamides, polyurethane and elastane-containing materials, and multicomponent textiles indicates that these outcomes arise from the coupled and time-dependent effects of polymer morphology, transport accessibility, supramolecular interactions, chemical reactivity, textile architecture, and process conditions. Selectivity should therefore not be regarded as an intrinsic property of either the DES or the polymer alone.

This mechanistic perspective also requires careful distinction between swelling, dissolution, and degradation. Changes in mass, fibre appearance, or crystallinity may indicate interaction with the medium, but they do not independently demonstrate molecular dissolution or polymer-chain scission. Reliable interpretation requires complementary evidence concerning morphology, crystallinity, chemical structure, molar mass, and reaction products.

The framework proposed here should be understood as a comparative interpretive tool rather than as a universal predictive model. Its practical application requires validation using real multicomponent textile waste together with assessment of recovered-product purity, DES regeneration and reuse, energy demand, safety, and scale-up. Progress beyond empirical solvent screening will therefore depend on matching DES composition and process conditions with polymer morphology, textile architecture, and the intended recovery pathway for each material fraction.

## Figures and Tables

**Table 1:** Conceptual shift from the conventional interpretation of DES/polymer interactions to the mechanistic perspective proposed in this review.

Conventional View	Mechanistic Perspective
Solvent–polymer affinity	Coupled transport–interaction phenomena
Dissolution-dominated process	Coupled swelling–dissolution–degradation behaviour
Uniform polymer accessibility	Morphology-dependent accessibility
Static solvent/polymer interactions	Dynamic structural evolution
Dissolution efficiency	Diffusion and swelling kinetics
Model polymer systems	Heterogeneous textile architectures
Strong solvent systems	Controlled selective destabilization

**Table 2:** Main limitations of current textile recycling strategies for selective separation of multicomponent textile waste.

Recycling Route	Primary Purpose	Main Limitation for Multicomponent Textile Waste	Relevance to Selective Separation	Representative References
Mechanical recycling	Recovery and reuse of fibres or textile fragments	Requires sorting and relatively homogeneous feedstock; fibre shortening and quality loss	Limited	[5,6,7,29,35]
Chemical depolymerization	Conversion of selected polymers to monomers or oligomers	Requires control of polymer-specific reactivity, product purification, and preservation of coexisting components	Moderate to high, system-dependent	[11,12,13,35,36,37,38]
Solvent-based separation	Selective dissolution and recovery of polymer fractions	Solvent recovery, polymer purity, diffusion limitations and incomplete penetration	High potential, strongly system-dependent	[35,40,41]
Thermochemical conversion	Conversion to lower-molecular products or fuels	Loss of polymer-specific material value and low component selectivity	Low	[29,30,35]
Enzymatic/biological approaches	Selective depolymerization under mild conditions	Slow kinetics, limited polymer accessibility and enzyme-related constraints	Promising but emerging	[29,35]

**Table 3:** Key physicochemical properties of deep eutectic solvents relevant to textile recycling.

DES Property	Main Compositional or Process Variables	Relevance for Textile Recycling	Key References
Supramolecular organization	HBA/HBD identity, molar ratio, water content, temperature	Governs the availability of interaction sites and the overall solvent environment	[20,21,22,23]
Viscosity	Component identity, molar ratio, temperature, water content	Influences solvent handling, impregnation, mass transfer and penetration into textile structures	[20,21,23]
Polarity and hydrophobicity	Chemical nature of DES components	Affects compatibility with hydrophilic and hydrophobic polymer domains	[20,21,23,26]
Hydrogen-bonding ability	Type and concentration of HBA/HBD components	Determines interactions with hydroxyl, amide, urethane and ester-containing polymer systems	[20,21,22,23]
Acid–base and Lewis-acid character	Acidic/basic HBDs, metal-containing components, additives	Can promote selective bond activation, hydrolysis or catalytic depolymerization	[10,20,44]
Water content	Controlled addition or uptake of water	Can reduce viscosity but also reorganize the DES network and alter solvation and reactivity	[21,23]
Thermal stability and liquid range	DES composition and phase behaviour	Defines the accessible processing window and solvent recovery conditions	[20,21]
Regeneration and process compatibility	Phase behaviour, miscibility, product separation strategy	Determines whether selective interaction can be translated into a practical recycling process	[10,20,26]

**Table 4:** Influence of polymer morphology on transport accessibility and selective behaviour in DES systems.

Structural Feature	Main Effect on Transport Accessibility	Potential Implication During DES Treatment	Representative References
High crystallinity and chain orientation	Reduced free volume and slower solvent penetration	Surface-limited interaction; delayed structural response	[31,32,33,34,50,53,56,57,58,59,60]
High amorphous fraction and segmental mobility	Greater accessibility of polymer segments	Faster solvent uptake and easier local reorganization	[31,32,33,34,50,51,52,53]
Skin-core or radial fibre heterogeneity	Non-uniform diffusion across the fibre cross-section	Different response of surface and inner fibre regions	[35]
Surface finishes, coatings and laminates	Restricted direct contact between DES and polymer matrix	Delayed or incomplete penetration into the polymer phase	[35,40,61]
Local defects, ageing and degradation-damaged regions	Locally increased permeability	Preferential solvent ingress and heterogeneous response	[35,61]
Multilayer textile architecture and mixed fibre assemblies	Complex diffusion pathways and phase-specific accessibility	Variable response of individual polymer components	[35,40,61]

**Table 5:** Experimental indicators for distinguishing swelling, dissolution and degradation in DES/polymer systems.

Observed Phenomenon	Minimum Supporting Methods	Interpretation Supported by the Evidence	What Cannot Be Concluded from This Observation Alone	Representative Literature
Increase in sample mass or volume	Gravimetry, optical microscopy; ideally SEC/GPC of recovered polymer	*Swelling* or solvent uptake	Molecular dissolution or chemical degradation	[55,67]
Local softening, increased transparency or fibre expansion	Optical microscopy, DSC, rheology	Partial swelling or local supramolecular destabilization	Complete bulk penetration or chain scission	[55,66]
Decrease in crystallinity without substantial molar-mass loss	DSC, XRD/WAXS combined with SEC/GPC	Decrystallization or structural reorganization	Molecular dissolution or degradation	[55,67,68]
Surface roughening, cracking or erosion	SEM, optical microscopy, mass balance	Surface-limited interaction or heterogeneous destabilization	Molecular dissolution of the whole polymer phase	[55,66]
Homogeneous solution after removal of insoluble residues	Filtration, polymer regeneration, rheology, SEC/GPC	Molecular dissolution, provided that polymer chains are present in the liquid phase	Preservation of molar mass or absence of concurrent degradation	[15,40]
Reduced molar mass, altered viscosity, oligomer or monomer formation	SEC/GPC, rheology, HPLC/GC, NMR	Chemical degradation or depolymerization	Whether prior swelling or dissolution was absent	[8,14,44,65]
Loss or formation of characteristic chemical groups	FTIR, Raman, NMR	Chemical modification or chain cleavage	Complete polymer conversion or homogeneous dissolution	[8,65]
Morphological change without clear molecular-weight decrease	Combined microscopy, DSC/XRD and SEC/GPC	Swelling or supramolecular reorganization rather than confirmed degradation	Chemical chain scission	[55,67,68]

**Table 6:** Main factors determining selective behaviour in DES/polymer systems.

Factor Category	Representative Factors	Primary Role During DES–Polymer Interaction	Possible Consequence for Selective Behaviour	Representative References
Polymer-related	Crystallinity, chain orientation, amorphous fraction, free volume, Tg, functional groups	Determines transport accessibility, segmental mobility and accessible interaction sites	Surface-limited response, delayed swelling, preferential interaction with amorphous domains or different chemical reactivity	[31,32,33,34,35,50,51,52,53,56,57,58,59,60]
DES-related	HBA/HBD composition, molar ratio, polarity, viscosity, hydrogen-bonding ability, acidity/basicity, Lewis-acid character, water content	Determines solvent structure, transport properties and interaction with polymer functional groups	Different extent of swelling, dissolution, bond activation or depolymerization	[20,21,22,23,24,25,26,44,69,70,71]
Process-related	Temperature, time, DES/polymer ratio, reagent/polymer ratio, agitation, particle size, microwave or ultrasound input	Controls transport kinetics, reaction rate and residence time of the polymer in the reactive medium	Transition from partial interaction to more extensive dissolution or degradation; changes in product selectivity	[8,14,15,16,44,62,70,72,73]
Textile-related	Fibre blend composition, multilayer architecture, elastane, coatings, dyes, contamination, ageing and degradation state	Creates heterogeneous transport pathways and component-specific accessibility	Different response of individual fibres, incomplete separation or altered purity of recovered fractions	[5,6,29,30,35,40,61,66,74]

**Table 7:** Representative DES-based approaches for cellulose-based fibres and cotton-containing textile systems.

DES System	Material	Reported Process/Main Observation	Cautious Mechanistic Interpretation	Ref.
ChCl/imidazole + PEG	Cellulose	Enhanced cellulose dissolution; reduced crystallinity of regenerated cellulose	Improved accessibility and disruption of supramolecular organization	[68]
ChCl/oxalic acid	Cellulose	Highest cellulose solubility among tested ChCl-based systems	Combined hydrogen-bonding interactions and favourable transport properties	[67]
ChCl/citric acid	Cellulose	Intermediate cellulose solubility	Partial disruption of cellulose interactions	[67]
ChCl/urea or ChCl/glycerol	Cellulose	Lower solubility than acidic ChCl-based DES	Less favourable balance of interaction strength and transport properties	[67]
ChCl/oxalic acid·2H_2_O + microwave/ultrasound	Cotton fibres	Cellulose nanocrystal production; 74.2% yield under optimized conditions	H-bond cleavage and structural accessibility increase rather than simple dissolution	[55]
ChCl/TsOH	PET/cotton textile	Cotton degradation with recovery of PET, MCC and glucose	Acid-assisted cellulose destabilization and partial hydrolytic degradation	[79]
ChCl/lactic acid	Waste PET/cotton textile	Separation into high-purity cotton and PET fractions; PET retained	Selective fractionation with partial modification of the cotton fraction	[80]
ZnCl_2_/AlCl_3_/H_2_O	PET/cotton/PU textile	Selective cotton dissolution; PET and PU retained in solid phase	Coordination- and hydrogen-bond-assisted disruption of cellulose network	[66]
ChOH/urea/zinc glycinate	Microcrystalline cellulose	12 wt% cellulose dissolved at 65 °C within 60 min; >80% solubilization efficiency retained after three reuse cycles	High dissolution capacity with demonstrated reuse potential; transferability to oriented textile fibres remains unverified	[49]

**Table 8:** Representative DES-based approaches for polyester fibres and PET-containing textile systems.

DES System/Strategy	Material	Reported Process and Main Result	Cautious Mechanistic Interpretation	Ref.
K_2_CO_3_:ethylene glycol	PET	Glycolysis; 100% PET conversion and 88% BHET yield under optimized conditions	Base-assisted DES-catalyzed glycolysis; performance depends on composition and process conditions	[44]
ChCl:Zn(OAc)_2_	Post-consumer PET	Glycolysis; >99% PET conversion and 91.6% BHET selectivity	Zn-containing DES promoted glycolysis; H-bonding and coordination interactions were proposed	[70]
KF:ethylene glycol (1:6)	Post-consumer PET flakes	Quantitative conversion at 180 °C within 15 min; five reuse cycles	Rapid fluoride-assisted glycolysis; water affects selectivity and stability	[84]
Betaine:Zn(OAc)_2_	PET/cotton textile	Complete PET conversion; 85% BHET yield; 95% cotton recovery	Selective glycolytic depolymerization of PET while retaining most cotton fibres	[14]
ChCl:ethylene glycol + NaOH	PET/cotton textile	Complete PET degradation; 98.95% TPA yield; <3% cotton mass loss	DES-assisted alkaline hydrolysis of PET	[8]
ChCl:glycerol + NaOH + microwave	PET/cotton textile	Complete reported PET depolymerization within 80–100 s	Combined effect of alkaline medium, DES and microwave heating; individual contributions not fully separable	[62]
Menthol:benzoic acid	PET/cotton textile	Reported complete PET dissolution; 97% PET recovery and 100% cotton recovery	Selective PET dissolution and polymer recovery without intended depolymerization	[15]

**Table 9:** Representative DES-related and benchmark approaches for polyamide fibres.

DES System/Strategy	Material	Reported Process and Main Result	Cautious Mechanistic Interpretation	Ref.
FeCl_3_·6H_2_O:acetic acid	PA66 pellets and fibres	Quantitative PA66 conversion at 180 °C for 5 h; monomer recovery after work-up	Lewis/Brønsted acid-assisted hydrolytic depolymerization of amide bonds	[88]
FeCl_3_·6H_2_O:methanesulfonic acid	Post-consumer PA66 nylon hosiery	Quantitative conversion and recovery of adipic acid and hexamethylenediamine-derived salt reported	Acidic DES-enabled depolymerization of real nylon textile waste	[88]
ChCl:monoethanolamine with DMAP	Discarded PA6 fishing nets	Approximately 80% conversion at 156 °C after 2 h	DES-assisted solvolysis; chain scission inferred from ATR-FTIR, product distribution not yet fully resolved	[89]
tert-Amyl alcohol + catalytic KOH *	Nylon/elastane textiles	Elastane selectively removed; polyamide-containing residual fabric retained	Selective elastane solvolysis with preservation of the polyamide phase	[90]

* Non-DES benchmark system included for mechanistic comparison.

**Table 10:** Representative DES-based approaches for polyurethane and elastane systems.

DES System/Strategy	Material	Reported Process and Main Result	Cautious Mechanistic Interpretation	Ref.
ChCl:urea (1:2)	Polycarbonate-based PU elastomer	100% PU degradation at 170 °C for 8 h; 57.4% PCDL yield	Selective cleavage of carbamate and urea bonds with limited carbonate-bond degradation under the reported conditions	[93]
ChCl:urea + ultrasound	Thermoplastic polyurethane	At 150 °C, degradation increased from 58.51% to 63.71% with ultrasonication; GPC, FTIR and NMR indicated chain scission	Ultrasonication enhanced DES-assisted chemical degradation; individual transport and chemical contributions remain coupled	[72]
Lactic acid:ZnCl_2_ (1:1) + white light	Polyurethane	Approximately 70 wt.% dissolution at 60 °C; regenerated material had lower molar mass and altered functional groups	Combined dissolution and depolymerization with evidence of urethane-bond cleavage	[65]
MeBA and MeLA DES	PET/EL, PA6/EL and PA66/EL textiles	Complete EL removal within 10–20 min; five reuse cycles; matrix polymers re-extruded	Selective EL dissolution with preservation of PET and PA fibres; blend-dependent process window	[95]
ZnCl_2_/AlCl_3_/H_2_O DES followed by DMSO	PET/cotton/PU textile	DES selectively removed cotton; PU was subsequently separated from PET using DMSO	PU remained stable during the first DES step; this is evidence for staged separation, not direct DES dissolution of PU	[66]

**Table 11:** Representative selective separation strategies reported for multicomponent textile systems.

Textile System	Targeted Component	Medium/Strategy	Reported Outcome	Cautious Interpretation	Ref.
PET/cotton	PET	Menthol:benzoic acid	PET dissolved and regenerated; cotton retained	Selective PET dissolution with polymer recovery	[15]
PET/cotton	PET	Betaine:Zn(OAc)_2_ + ethylene glycol	PET glycolysis; cotton recovered	Selective PET depolymerization rather than polymer dissolution	[14]
PET/cotton	PET	ChCl:ethylene glycol + NaOH	PET hydrolysis; cotton largely preserved	DES-assisted alkaline degradation of PET	[8]
PET/cotton	PET	ChCl:glycerol + NaOH + microwave heating	Rapid removal of PET from blend fabrics	Combined microwave-assisted alkaline depolymerization; individual effects remain coupled	[62]
PET/cotton	Cotton	ChCl:TsOH	Cotton degradation and PET recovery	Acidic DES-mediated transformation of the cellulosic phase	[79]
PET/cotton	Cotton	ZnCl_2_/H_3_PO_4_/H_2_O	Cotton dissolved; PET retained	Selective cellulose dissolution in a metal-salt hydrate system	[16]
PET/EL, PA6/EL and PA66/EL	Elastane	MeBA or MeLA DES	Complete EL removal; matrix polymers preserved and re-extruded	Selective EL dissolution with a blend-dependent process window	[95]
PET/cotton/PU	Cotton, then PU and PET	ZnCl_2_/AlCl_3_/H_2_O DES followed by DMSO and PET depolymerization steps	Sequential recovery of all three components	Multistep, phase-specific separation; only the first step is DES-based	[66]

**Table 12:** Synthesis of representative experimental observations supporting a common mechanistic interpretation of DES–polymer interactions in textile systems.

Polymer or Textile System	Primary Structural or Transport Constraint	Evidence-Supported Response	Mechanistic Implication	Representative References
Cellulose and cotton	Hydrogen-bond network, fibrillar morphology and crystalline domains	Swelling, decrystallization, dissolution or acid-assisted degradation depending on DES formulation and conditions	Structural accessibility and progressive disruption of hydrogen bonds must be distinguished from confirmed chain degradation	[55,66,67,68,79]
PET and polyester textiles	Semicrystalline morphology, chain orientation and accessibility of ester bonds	Surface activation, alkaline hydrolysis, glycolysis or reported selective dissolution	The final product determines whether the process is modification, depolymerization or polymer recovery	[8,14,15,44,62,70,71,81,82,83]
Polyamides	Amide hydrogen bonding, crystalline structure and moisture-sensitive amorphous regions	DES-assisted solvolysis or hydrolytic depolymerization in reactive acidic systems	Current evidence supports chemical transformation in selected systems, but direct textile DES studies remain limited	[88,89]
Polyurethane and elastane-containing systems	Segmented morphology, soft/hard domains, urethane-bond reactivity and blend-dependent accessibility	Chemical chain cleavage in model PU systems; selective elastane dissolution from PET/EL and PA/EL textiles with preservation of matrix fibres	PU depolymerization and elastane dissolution represent distinct pathways; the companion polymer and textile architecture determine the process window	[65,66,72,93,95]
Multicomponent textile waste	Fibre architecture, coatings, dyes, elastane, contamination and phase-specific accessibility	Sequential or component-specific separation, often using more than one processing step	Selectivity must be assessed for the entire textile system, including purity and integrity of every recovered fraction	[8,14,15,62,66,79]

## Data Availability

No new data were created or analyzed in this study. Data sharing is not applicable to this article.

## Abbreviations

The following abbreviations are used in this manuscript:

**Abbreviation**	**Definition**
ATR-FTIR	Attenuated total reflectance Fourier-transform infrared spectroscopy
BHET	Bis(2-hydroxyethyl) terephthalate
ChCl	Choline chloride
ChOH	Choline hydroxide
DES	Deep eutectic solvent
DMAP	4-(Dimethylamino)pyridine
DMSO	Dimethyl sulfoxide
DSC	Differential scanning calorimetry
EG	Ethylene glycol
EL	Elastane
FTIR	Fourier-transform infrared spectroscopy
GC	Gas chromatography
GPC	Gel permeation chromatography
HBA	Hydrogen-bond acceptor
HBD	Hydrogen-bond donor
HPLC	High-performance liquid chromatography
LTTM	Low-transition-temperature mixture
MALDI-TOF-MS	Matrix-assisted laser desorption/ionization time-of-flight mass spectrometry
MCC	Microcrystalline cellulose
MEA	Monoethanolamine
MeBA	DL-Menthol:benzoic acid deep eutectic solvent
MeLA	DL-Menthol:lauric acid deep eutectic solvent
NADES	Natural deep eutectic solvent
NMMO	N-Methylmorpholine N-oxide
NMR	Nuclear magnetic resonance spectroscopy
PA	Polyamide
PA6	Polyamide 6
PA66	Polyamide 6,6
PCDL	Polycarbonate diol
PEG	Polyethylene glycol
PET	Poly(ethylene terephthalate)
PU	Polyurethane
SEC	Size-exclusion chromatography
SEM	Scanning electron microscopy
TGA	Thermogravimetric analysis
TPA	Terephthalic acid
TPU	Thermoplastic polyurethane
TsOH	*p*-Toluenesulfonic acid
WAXS	Wide-angle X-ray scattering
XRD	X-ray diffraction
[DBNH][OAc]	1,5-Diazabicyclo[4.3.0]non-5-enium acetate
